# Safety and pharmacokinetics of sulfasalazine and its metabolite sulfapyridine for treatment of preterm preeclampsia in Australia (SIP): an early phase, unblinded, single-arm, proof of concept clinical trial

**DOI:** 10.1016/j.eclinm.2026.103779

**Published:** 2026-02-03

**Authors:** Elif Kadife, Eric Decloedt, Vanessa Louw, Zohra Bibi Abdulla, Tracy Kellermann, Veshni Pillay-Fuentes Lorente, Catherine Cluver, Anna Middleton, Tu'uhevaha J. Kaitu'u-Lino, Joanne M. Said, Stephen Tong, Fiona C. Brownfoot

**Affiliations:** a Obstetrics Diagnostics and Therapeutics Group, Department of Obstetrics, Gynaecology and Newborn Health, University of Melbourne, Melbourne, Victoria, Australia; b Mercy Perinatal, Mercy Hospital for Women, 163 Studley Road, Heidelberg, Melbourne, Victoria, Australia; c Division of Clinical Pharmacology, Department of Medicine, Stellenbosch University, Tygerberg Hospital, 7505, Cape Town, South Africa; d Department of Obstetrics and Gynaecology, Stellenbosch University, Tygerberg Hospital, 7505, Cape Town, South Africa; e Maternal Fetal Medicine, Joan Kirner Women's & Children's at Sunshine Hospital, Western Health and Department of Obstetrics Gynaecology and Newborn Health, University of Melbourne, Australia

**Keywords:** Sulfasalazine, Preeclampsia, Pharmacokinetics

## Abstract

**Background:**

Preeclampsia is a hypertensive pregnancy disorder marked by systemic inflammation and endothelial dysfunction. Sulfasalazine, an anti-inflammatory agent, may have therapeutic potential in preeclampsia. This study investigated its pharmacokinetics, placental transfer, and safety profile of sulfasalazine and its metabolite, sulfapyridine in women with preterm preeclampsia.

**Methods:**

A prospective, open-label pharmacokinetic trial was conducted at two hospitals in Melbourne, Australia, (February 2018–December 2020). Participants between 24+0- and 36+0- weeks’ gestation with a singleton, non-anomalous pregnancies and a diagnosis of preeclampsia were included received 3 g/day of oral sulfasalazine. Serial maternal blood samples were collected post-dose. Maternal and umbilical cord blood and placental tissue were collected at birth. Primary outcomes were maternal–fetal safety and pharmacokinetics. Maternal plasma levels of soluble fml-like tyrosine kinase-1 (sFlt-1) and placental growth factor (PlGF) were measured. All participants receiving at least one dose were included in analyses. The trial was prospectively registered with ANZCTR (12617000226303).

**Findings:**

Twelve participants were enrolled, seven had pharmacokinetic sampling. No serious adverse events occurred relating to sulfasalazine use. Sulfasalazine and its metabolite sulfapyridine were detected in maternal plasma, placenta and in the fetal circulation. Maternal-to-fetal transfer of both compounds was confirmed, with some participants showing higher concentrations in cord blood than maternal blood, suggesting potential active or saturable transport mechanisms. Antiangiogenic biomarkers, including sFlt-1, increased post-treatment in most participants.

**Interpretation:**

Sulfasalazine was well tolerated in women with preterm preeclampsia and showed maternal and fetal exposure. Placental transfer was evident. These findings support the feasibility of sulfasalazine use in this population and warrant further investigation in a larger clinical trial to evaluate efficacy.

**Funding:**

10.13039/100015988Norman Beischer Medical Research Foundation, 10.13039/501100001782University of Melbourne, and 10.13039/501100000925NHMRC. Funders had no role in study conduct or manuscript preparation.

**Research in context**

**Evidence before this study**

We searched online medical databases, including PubMed and Embase, to identify previous studies investigating the use of sulfasalazine during pregnancy, particularly in women with preeclampsia. The search covered studies published from December 2000 until 2023, using terms such as “sulfasalazine,” “pregnancy,” “preeclampsia,” “placenta,” and “pharmacokinetics.” We included both clinical and laboratory studies without language restrictions. Most previous research focused on the safety of sulfasalazine in pregnancy for chronic conditions like inflammatory bowel disease, but little is known about its pharmacokinetics in pregnant women with preeclampsia or its effects on maternal or fetal biomarkers related to the disease. No clinical trials were found that evaluated the transfer of sulfasalazine or its metabolites to the placenta or fetus in the context of preeclampsia.

**Added value of this study**

This is the first study to examine the pharmacokinetics of sulfasalazine and its active metabolite sulfapyridine in women with preterm preeclampsia. We demonstrate that sulfasalazine is present in maternal and fetal blood, as well as in the placental tissue. This study also shows how sulfasalazine may influence biological markers that are altered in preeclampsia, without causing any observable harm to mothers or babies. Our findings offer early insights into how this well-known anti-inflammatory drug behaves in a pregnancy complicated by preeclampsia.

**Implications of all the available evidence**

This study lays the foundation for future clinical trials exploring sulfasalazine as a potential treatment for preeclampsia. It confirms that the drug reaches maternal, fetal, and placental compartments and appears to be safe in a small group of participants. These findings suggest that with further research, sulfasalazine could be repurposed to help treat preeclampsia and improve outcomes for both mothers and their babies.

## Introduction

Preeclampsia is a multisystem disorder affecting 5% of pregnant women and is a major cause of maternal and perinatal mortality and morbidity.^1^ It is characterised by hypertension with liver, renal, hematologic or neurological involvement and can also impact the fetus with fetal growth restriction. It stems from placental dysfunction that causes an imbalance in pro- (Placental Growth Factor (PlGF)) and anti- (Soluble fms-like tyrosine kinase-1 (sFlt-1)) angiogenic factors.^2^ This causes widespread endothelial cell dysfunction that manifests clinically as hypertension and multisystem organ involvement after 20 weeks of gestation. There is currently no disease-modifying pharmacotherapy, and delivery is the only way to stop disease progression. This causes prematurity in the baby if birth is needed prior to 37 weeks.^3^ There is an urgent need for effective pharmacotherapies for preeclampsia.

Sulfasalazine, a well-known disease-modifying drug commonly used to treat inflammatory bowel disease and rheumatoid arthritis, has been investigated for its potential therapeutic benefits in preeclampsia due to its anti-inflammatory properties.^4^^,^^5^ Sulfasalazine is generally well tolerated and has not been associated with an increased risk of congenital fetal abnormalities.^4^, ^5^, ^6^, ^7^, ^8^

Encouragingly, it has been shown to be effective in reducing key features of preeclampsia in human placental models, including lowering anti-angiogenic factors like sFlt-1, reducing oxidative stress, and improving endothelial function.^4^^,^^6^ These mechanistic effects provide the rationale for its investigation in clinical trials.

Sulfasalazine is primarily metabolised by intestinal bacteria into two major active metabolites: sulfapyridine, which is systemically absorbed, and 5-aminosalicylic acid, which largely remains in the gut. Less than 15% of the parent drug is absorbed in the small intestine and reaches systemic circulation.^9^^,^^10^ Limited pharmacokinetic data suggest that sulfasalazine can cross the placenta, with umbilical cord concentrations similar to maternal concentration.^10^ However, there is no pharmacokinetic data specific to preeclampsia. Given that sulfasalazine is more than 99% protein-bound, the hypoalbuminemia and altered volume of distribution associated with preeclampsia may significantly impact its pharmacokinetics. Furthermore, the most common adverse event of sulfasalazine is gastrointestinal and tolerability data on the use of sulfasalazine during preeclampsia are lacking.

We aimed to examine the pharmacokinetics of oral sulfasalazine and its metabolite sulfapyridine, assessing their concentrations in maternal blood, placental tissue and concentrations within the cord blood prior to conducting clinical trials exploring efficacy as a treatment for preeclampsia. We also assessed the safety and tolerability in participants with preterm preeclampsia. We explored the effect of sulfasalazine on pro- and antiangiogenic factors in the maternal serum.

## Methods

### Study design

We conducted a prospective, open-label, pharmacokinetic clinical trial to assess the pharmacokinetics of sulfasalazine in pregnant women diagnosed with preterm preeclampsia. The trial was conducted from February 2018 to December 2020 at the Mercy Hospital for Women and Sunshine Hospital in Melbourne, Australia. The trial protocol was approved by the Mercy human ethics committee (HREC #R16/65), and written informed consent was obtained from all participants. The trial was registered with Australia and New Zealand Clinical Trials Registery (ANZCTR) (identifier 12617000226303).

Initially, a planned sample size of 20 participants was chosen based on feasibility considerations, with the aim of generating preliminary pharmacokinetic and safety data sufficient to inform dose selection and study design for future trials. However, only 12 women were ultimately recruited. This shortfall reflects the inherent difficulties of enrolling acutely unwell women with preeclampsia, many of whom required urgent delivery soon after diagnosis, limiting the window for recruitment. Despite this, the number of participants enrolled provided adequate pharmacokinetic and safety data to meet the exploratory objectives of this early-phase study.

### Participants

The diagnosis of preeclampsia was made using the Society of Obstetric Medicine Australia and New Zealand guidelines^11^ and participants meeting the following criteria were approached for study participation: hypertension (SBP ≥140 mmHg and/or DBP ≥90 mmHg) after 20 weeks' gestation with involvement of one or more organ systems, including renal, hepatic, hematologic, neurological, or fetal (Table 1). Inclusion criteria were women aged 18 years or older, with singleton, non-anomalous pregnancies between 24 + 0 and 36 + 0 weeks of gestation, and willing to provide written informed consent. Exclusion criteria included: multiple pregnancies, known fetal malformations, contraindications to sulfasalazine, current use of sulfasalazine, or presence of immunodeficiency disorders. Sex/gender data were self-reported by participants.

**Table 1:** Participant baseline characteristics (n = 7).

Characteristic	Sulfasalazine (N = 7)
Race^a^	
White	6
Asian	1
Age (years), median (IQR)	32 (29.5–37)
Body mass index (kg/m^2^), median (IQR)	24.6 (22.3–26.6)
Obesity^b^	1
Gravity, median (IQR)	2 (1–4)
Parity, median (IQR)^c^	0.5 (0–1)
Gestational age at recruitment (weeks), median (IQR)	31.85 (30.28–35.14)
Gestational age at delivery (weeks), median (IQR)	33.42 (33–36.85)
Chronic hypertension	3
Asthma	2
Use of low-dose aspirin	1

**IQR**: Interquartile range.

a Race and ethnicity were self-reported by participants.

b Obesity is defined as BMI ≥30 kg/m2 using pre-pregnancy weight.

c Parity is any pregnancy that lasted >20 weeks.

### Randomisation and masking

As this was an early-phase, open-label pharmacokinetic study, there was no randomisation or blinding. All participants received the same intervention (sulfasalazine), and both participants and investigators were aware of treatment allocation.

### Procedures

Participants were admitted to hospital and administered sulfasalazine (Traded as: Salazopyrin EN Pfizer Australia Pty. Ltd., Australian Register of Therapeutic Goods ID 14485) orally in divided (every 12 h) doses totalling 2–3 g per day. Dosing was commenced at 1.5 g 12 hourly and reduced to 1 g 12 hourly if poorly tolerated due to side effects. In those with dose reductions, symptoms subsided with lower doses, which were not subsequently increased. Adherence was confirmed by direct observation by clinical staff.

Pharmacokinetic sampling was performed after steady state was achieved, which in our protocol was pragmatically defined as more than 30 h following commencement of sulfasalazine, consistent with the known half-life of its metabolite sulfapyridine (∼7–15 h). Maternal blood samples were collected at 0, 2, 4-, 8-, 12- hours post-dose in pink topped plasma EDTA tubes. Maternal and umbilical cord blood and placenta samples were collected at delivery to determine sulfasalazine and sulfapyridine concentrations. Plasma drug concentrations were measured by liquid chromatography tandem mass spectrometry (LC-MS), together with placental drug and metabolite concentrations, as previously described.^12^ Sulfasalazine and sulfapyridine concentrations reported in this study represent total drug (protein-bound + unbound); unbound (free) concentrations were not measured.

Clinical care, including decisions around delivery timing, was at the discretion of the treating clinical team. The hospitals' local Standard Operating Procedures was used in managing preterm preeclampsia (including blood tests ≥3 times per week; twice weekly growth and wellbeing ultrasounds; umbilical artery Doppler and CTG monitoring 3 times per week)–or management as otherwise ordered by the treating obstetric unit. A medically qualified investigator assessed the cause, severity and likelihood of adverse events being related to the IMP, referenced against the safety and toxicity information detailed in the manufacturer's Product Information.

All clinical data were collected or abstracted by trained research coordinators at each site and entered a central database managed by the data coordinating centre responsible for data analysis.

### Outcomes

The primary outcome was to evaluate the safety and pharmacokinetics of sulfasalazine and sulfapyridine in women with preterm preeclampsia. Pharmacokinetic variables included the area under the concentration–time curve (AUC) between 0 and 12 h, maximum plasma concentration (C_max_), time to reach maximum concentration (T_max_) with paired maternal plasma and umbilical cord plasma concentrations.

The co-primary safety outcomes included maternal monitoring for adverse events. Interval fetal wellbeing assessments included twice-weekly biophysical profiles, amniotic fluid indices, and Doppler studies.

Secondary outcomes included maternal outcomes, (including gestational age at delivery, duration of therapy, maternal conditions, blood pressure, antihypertensive and aspirin use, adverse events, and hospital stay). Neonatal outcomes included birthweight, centile, reason for delivery, respiratory distress syndrome, neonatal intensive care admission (>48 h), and level of care required. Birthweight centiles were calculated by deriving a z-score for each neonate:

$$z=\frac{\text{Observed}\;\text{Birthweight}-\mu_{\text{GA},\text{sex}}}{\sigma_{\text{GA},\text{sex}}}$$

where μ and σ represent the mean and standard deviation of birthweight at the corresponding gestational age and sex from the Australian reference dataset.^13^ The z-score was then converted to a centile using the standard normal distribution.

Biomarker analysis measuring maternal plasma soluble fms-like tyrosine kinase-1 (sFlt-1) and placental growth factor (PlGF) levels were done using a commercial electrochemiluminescence immunoassay (Roche Diagnostics).

### Statistical analysis

Clinical variables and the clinical course were presented as a descriptive statistics. Steady state sulfasalazine and sulfapyridine pharmacokinetic parameters were estimated using non-compartmental analysis conducted with PKanalix (PKanalix 2023R1, Simulations Plus, https://doi.org/10.5281/zenodo.11401684). A linear up-log down method was used to evaluate the AUC of sulfasalazine and sulfapyridine. Statistical analysis was performed using GraphPad Prism 6 (GraphPad Software, La Jolla, CA). A p < 0.05 was considered statistically significant.

### Role of the funding source

The funder of the study had no role in study design, data collection, data analysis, data interpretation, or writing of the report.

## Results

**Fig. 1:**
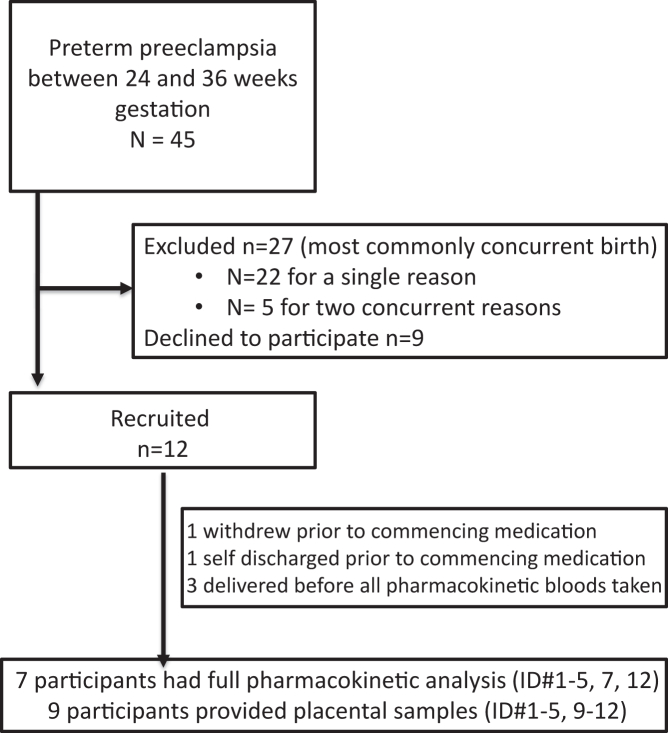
**Study recruitment and patient characteristics.** A total of 45 women with preeclampsia were screened for eligibility. Twenty seven women were excluded (18 were deemed to deliver within 48 h and 9 declined to participate). 12 women were recruited into the study, with 1 withdrawing prior to medication, 1 self-discharging before starting medication, and 3 delivering before all pharmacokinetic bloods were taken, these participants still provided placental samples (n = 9). Seven of them completed the full pharmacokinetic analysis.

A total of 45 women with preeclampsia were screened for eligibility between February 2018 to December 2020. Of the 45 women, 21 met the inclusion criteria, with 12 consenting to participate and 9 declining (Fig. 1). The remaining 27 women were excluded, with 22 excluded for a single reason and 5 excluded for two concurrent reasons, most commonly due to imminent birth in combination with another exclusion factor. Twelve women were recruited into the study, with 1 withdrawing prior to medication administration, 1 self-discharging before starting medication, and 3 delivering before all pharmacokinetic samples were taken (Fig. 1). Nine participants provided placental tissue for analysis and 7 completed full pharmacokinetic sampling. None of the 9 participants were lost to follow-up.

All participants who underwent full pharmacokinetic sampling received sulfasalazine at a dose of 1.5 g every 12 h, except one who required a dose reduction to 1 g twice daily after six days due to nausea. All participants continued sulfasalazine until delivery, except for one who was withdrawn by the researcher prior to birth due to worsening renal function, which was deemed unlikely to be related to the investigational product.

The baseline characteristics of the study participants in the sulfasalazine group are presented in Table 1 (n = 7). Two participants had a history of hypertension with one of them being essential hypertension with Graves’ disease. Only one of the seven participants reported the use of low-dose aspirin during pregnancy. Two participants reported having a diagnosis of asthma.

### Pharmacokinetics of sulfasalazine

**Fig. 2:**
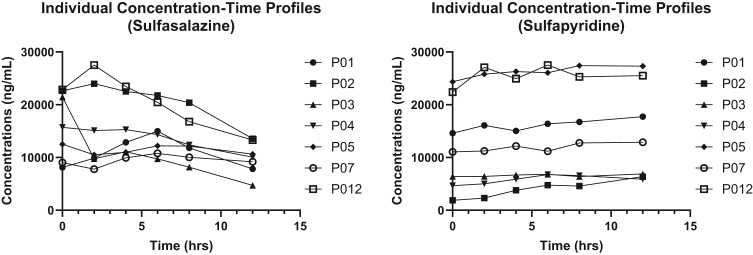
**Individual concentration–time plots for sulfasalazine and sulfapyridine in maternal plasma.** Blood samples were collected at 0, 2, 4-, 8-, 12-h post-dose to capture the pharmacokinetic profile of sulfasalazine.

The concentration-time profiles of sulfasalazine for individual participants are depicted in Fig. 2. Sulfasalazine had a mean terminal half-life of 12.7 ± 8.8 h (Table 2). The mean AUC_0-12_ was 164.46 ± 56.91 h⋅mg⋅L^−1^, with a coefficient of variation (CV) of 34.61%. Apparent oral clearance (CL/F) averaged 4.8 ± 2.9 L⋅h^−1^, and the volume of distribution (Vd/F) was 71.4 ± 25.5 L. The mean Cmax was 18.1 ± 6.3 mg⋅L^−1^, occurring at a median Tmax of 2 h (range: 0–6 h). The elimination rate constant (kel) was 0.072 ± 0.036 h^−1^, with high interindividual variability (CV 50%). Tlast was consistently measured at 12 h for all participants.

**Table 2:** Summary of sulfasalazine non-compartmental analysis (NCA) parameters.

	N	Mean	SD	CV(%)	Min	Median	Max
AUC_0-inf_ (h⋅mg⋅L^−1^)	5	379	151.68	40.02	152.41	411.57	567.09
AUC_0-12_ (h⋅mg⋅L^−1^)	7	164.46	56.91	34.61	114.31	137.54	246.47
AUC_0-12_/Dose (h⋅L^−1^)	7	0.11	0.038	34.61	0.076	0.092	0.16
AUC_0-tlast_ (h⋅mg⋅L^−1^)	7	164.46	56.91	34.61	114.31	137.54	246.47
CL/F (L⋅h^−1^)	5	4.82	2.88	59.75	2.65	3.64	9.84
C_last_ (mg⋅L^−1^)	7	9.9	3.09	31.23	4.71	10.07	13.56
C_max_ (mg⋅L^−1^)	7	18.13	6.25	34.47	10.78	15.71	27.5
T_1/2_ (h)	5	12.74	8.84	69.41	5.61	9.67	28.02
k_el_ (h^−1^)	5	0.072	0.036	50.01	0.025	0.072	0.12
T_last_ (h)	7	12	0	0	12	12	12
T_max_ (h)	7	2.29	2.69	117.7	0	2	6
V_d_/F (L)	5	71.38	25.5	35.72	44.37	77.06	106.92

Participants ID#1–5, 7, 12 provided maternal plasma samples. **AUC**: area under the plasma concentration–time curve; **AUC0-12**: area under the curve from 0 to 12 h; **AUC0-12/Dose**: dose-normalized area under the curve from 0 to 12 h; **AUC0-inf**: area under the curve from 0 extrapolated to infinity; **AUCall**: total observed area under the curve across all sampling points; **AUC0-tlast**: area under the curve from 0 to the last measurable concentration; **CL/F**: apparent clearance after oral dosing (clearance divided by bioavailability); **Clast**: observed concentration at the last measurable time point; **Cmax**: maximum observed plasma concentration; **CV**: coefficient of variation (100 × SD/mean); **kel**: elimination rate constant; **N**: number of participants included in the analysis; **SD**: standard deviation; **T1/2**: terminal half-life; **Tlast**: time of last measurable concentration; **Tmax**: time to reach maximum observed concentration (Cmax); **Vd/F**: apparent volume of distribution after oral dosing (volume of distribution divided by bioavailability).

Among the broader cohort (n = 7), mean C_max_ was 18.1 ± 6.3 mg⋅L^−1^, occurring at a median T_max_ of 2 h (range: 0–6 h). The elimination rate constant (k_el_) was 0.072 ± 0.036 h^−1^. T_last_ was consistently measured at 12 h for all participants. High variability was also observed for T_max_ (CV 117.7%), reflecting differences in absorption timing.

### Pharmacokinetics of sulfapyridine

Sulfapyridine had a mean AUC_0-12_ of 166.6 ± 111.4 h⋅mg⋅L^−1^ (CV = 66.9%) and AUC_0-12_ of 0.11 ± 0.074 h⋅L^−1^ (Table 3). Peak plasma concentrations (C_max_) averaged 15.1 ± 9.4 mg⋅L^−1^. The median time to peak concentration (T_max_) was 12 h (range 6–12 h), indicating slow absorption or conversion as it is a metabolite. Sulfapyridine concentrations reached a steady state and remained constant without a measurable terminal decline. As a result, terminal phase parameters such as kel, t_1_/_2_, CL/F, and Vd/F could not be estimated from the data.

**Table 3:** Summary of sulfapyridine non-compartmental analysis (NCA) parameters.

	N	mean	SD	CV(%)	min	median	max
AUC_0-inf_ (h⋅mg⋅L^−1^)	0						
AUC_0-12_ (h⋅mg⋅L^−1^)	7	166.59	111.38	66.86	50.18	144.27	317.53
AUC_0-12_/Dose (h⋅L^−1^)	7	0.11	0.074	66.86	0.033	0.096	0.21
AUC_all_ (h⋅mg⋅L^−1^)	7	166.59	111.38	66.86	50.18	144.27	317.53
AUC_0-tlast_ (h⋅mg⋅L^−1^)	7	166.59	111.38	66.86	50.18	144.27	317.53
CL/F (L⋅h^−1^)	0						
C_last_ (mg⋅L^−1^)	7	14.65	9.1	62.12	5.88	12.88	27.32
C_max_ (mg⋅L^−1^)	7	15.08	9.4	62.37	6.37	12.88	27.5
T_1/2_ (h)	0						
k_el_ (h^−1^)	0						
T_last_ (h)	7	12	0	0	12	12	12
T_max_ (h)	7	9.71	2.93	30.14	6	12	12
V_d_/F (L)	0						

### Placental sulfasalazine and sulfapyridine concentrations

Placental accumulation of sulfasalazine and its metabolite sulfapyridine was assessed in snap-frozen tissues collected from nine samples, including three for whom complete plasma pharmacokinetic data were not available (Table 4). Detectable concentrations of both compounds were observed in 7 out of 9 placentas, with 2 samples (APL_003 and APL_008) below the limit of quantification (BLQ). Among quantifiable samples, sulfasalazine concentrations ranged from 98.2 to 840 ng/mL, corresponding to tissue concentrations between 491 and 4201 ng/g. Placental tissue sulfapyridine concentrations ranged from 639 ng/g to 26,756 ng/g. The high metabolite-to-parent drug ratio suggests preferential accumulation of the metabolite within placental tissue. These findings confirm placental exposure and differential retention of sulfasalazine and its metabolite following maternal administration.

**Table 4:** Concentrations of sulfasalazine and sulfapyridine in snap frozen placenta samples.

ID	Mass of placenta (g)	Observed sulfasalazine (ng/ml)	Observed sulfapyridine (ng/ml)	Sulfasalazine (ng/g tissue)	Sulfapyridine (ng/g tissue)
P01	0.167	345	2294	1724	11,472
P02	0.124	294	172	1469	861
P03	0.147	BLQ	BLQ	BLQ	BLQ
P04	0.151	98.2	127	491	637
P05	0.127	253	2616	1267	13,078
P09	0.153	284	1421	1421	7104
P10	0.145	323	2207	1614	11,035
P11	0.136	BLQ	BLQ	BLQ	BLQ
P12	0.162	840	5351	4201	26,756

Participants ID#1–5, 9–12 provided placental samples. **BLQ:** Below limit of quantification.

### Individual concentration–time profiles for sulfasalazine and sulfapyridine

The concentration–time profiles of sulfasalazine and sulfapyridine for individual participants are depicted in Fig. 2. Peak plasma concentrations (C_max_) ranged from approximately 4706 ng/mL to 27,500 ng/mL and 1894 ng/mL to 27,500 ng/mL, respectively, between 0- and 12-h post-administration.

### Paired maternal and cord plasma concentrations of sulfasalazine

**Fig. 3:**
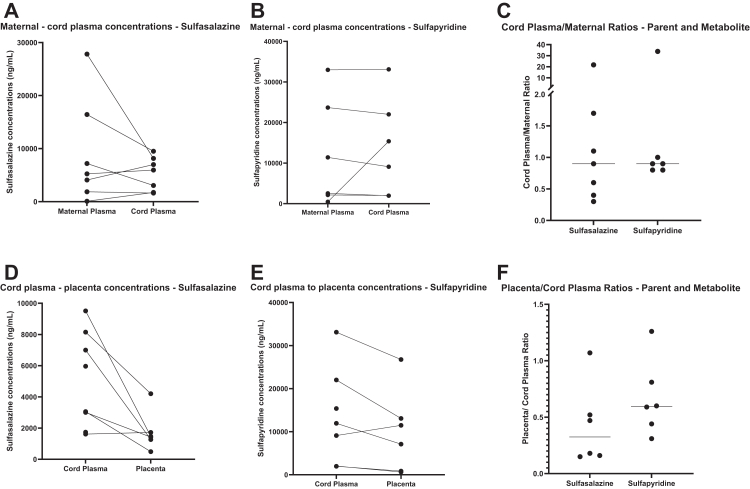
**Matched maternal and cord plasma collected at time of delivery to assess sulfasalazine and sulfapyridine concentrations.** Maternal-cord concentrations of **A)** Sulfasalazine. **B)** Sulfapyridine. **C)** Cord/Maternal ratio. Cord Plasma- Placenta concentration **D)** Sulfasalazine. **E)** Sulfapyridine. **F)** Placenta/Cord ratio. Maternal and cord concentrations are expressed as ng/mL (plasma), whereas placental concentrations are expressed as ng/g (tissue weight). Values are plotted on the same axis for visual comparison only; units are distinct.

Sulfasalazine distribution between maternal and cord plasma varied across participants: in three cases, maternal concentrations exceeded cord levels; in two, levels were comparable; and in the remaining two, cord levels were slightly higher (Fig. 3A). Maternal plasma concentrations had a median of 5246 ng/mL (IQR 1871–16,437), while cord plasma median concentration was 5964 ng/mL (IQR 1736–8152).

Median maternal active metabolite sulfapyridine concentrations were 6974 ng/mL (IQR 1725–26,006) (Fig. 3B). Cord plasma sulfapyridine concentrations were 12,243 ng/mL (IQR 2009–24,783) (Fig. 3B). In most cases, the matched cord concentrations reflect maternal levels, except for one participant where sulfapyridine was low in maternal blood (454 ng/mL) but exceeded 15,383 ng/mL in the cord (Fig. 3B).

The maternal to cord plasma ratios for both sulfasalazine and sulfapyridine are depicted in Fig. 3C. These ratios indicate that, on average, the ratios for both compounds are approximately 1, suggesting equal distribution between maternal and fetal blood.

Cord plasma to placenta concentration of both sulfasalazine (Fig. 3D) and sulfapyridine (Fig. 3E) were both reduced, however, ratio of the metabolite remained higher in the placenta (Fig. 3F). Placenta values are expressed in ng/g and plasma values in ng/mL; ratios therefore represent relative partitioning, not a unit-matched comparison.

### Serum levels of placental biomarkers

**Fig. 4:**
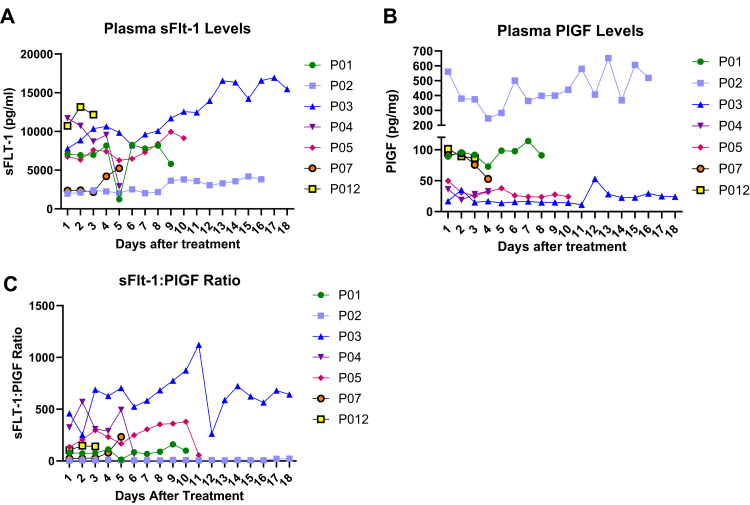
**Preeclampsia biomarker concentrations assessed days after treatment.** Maternal plasma concentrations of **A)** sFlt-1, **B)** PlGF, **C)** sFlt-1:PlGF ratio.

Plasma sFlt-1 levels increased over time (Fig. 4A). Overall, there are no discernible trends in the change in the ratios (Fig. 4C) and plasma levels do not appear to be altered in the days following treatment (Fig. 4A–B).

### Safety and tolerability

Adverse events were monitored throughout the study by medically qualified investigator (Table 5). Participants continued the investigational product until delivery without complications (n = 6). Three participants experienced mild nausea, one of whom required a dose reduction, while the other's symptoms resolved without treatment. Another participant was withdrawn from the study due to worsening renal function. One infant was admitted to the Special Care Nursery (SCN) following three episodes of transient dusky spells; however, on review this was not attributed to the investigational product. Other reported side effects included yellow urine (n = 1).

**Table 5:** Adverse and serious adverse events experienced by subjects, with causality assessment relative to study medication (n = 7).

Adverse event	Participants	Severity	Resolution	Related to product
Nausea				
	2	Mild	Resolved Without Medication	Possibly Related
	1	Mild	Required dose reduction, well tolerated at 2 g daily	Possibly Related
Serious adverse event (infant scn admission)	1	Serious	Not Attributed to Investigational Product	Not Related
Worsening renal function	1	Serious	Stopped IMP at day 12	Possibly Related
Yellow urine	1	Mild	No Further Complications Reported	Possibly Related

Consistent with the presence of preeclampsia fetal outcomes showed a high prevalence of small for gestational age (SGA) infants and fetal growth restriction (FGR) (Table 6). Birthweights ranged from 1,678 g to 2,910 g and the centiles from <5 to 50. Several cases exhibited oligohydramnios and abnormal fetal Dopplers, requiring close monitoring. Due to prematurity, most neonates required SCN or neonatal intensive care unit (NICU) admission. Common neonatal complications included respiratory distress syndrome (RDS), transient tachypnoea of the newborn (TTN), presumed sepsis, and low blood glucose levels. Some infants needed intravenous (IV) antibiotics and monitoring, though most complications had resolved within a few days. While these complications were not thought to be related to the investigational product, they do highlight the impact of hypertensive disorders and fetal growth restriction on maternal and neonatal outcomes.

**Table 6:** Fetal and Neonatal outcomes of study participants.

Gestational age at delivery	EFW %, AC %, AFI, UA	Reason for delivery	Fetal sex	Birth weight (g)	Birth weight centile (%)	Highest level of care	NICU/SCN stay
37.57	3%, AC 5%, AFI 8.1	Dusky episodes, low BGL, sepsis suspected	F	2240	4.6	SCN	3 days SCN
36.85	25%, AC 50%, AFI A, UA N	Labile blood pressures, (160/100)	F	2910	43.8	Ward	Nil
33.28	5%, AC 5%, AFI, UA N	–	M	1746	16.6	SCN	Yes
32.42	3%, AC 3%, AFI, UA N	Increasing BP, maternal ascites, TTN	F	1369	46.8	SCN	Yes
33	<3rd, AC <3rd, AFI oligo, UA N	intermittent absent EDF. Maternal disease stable.	F	1678	17.3	SCN	–
32.85	–	Blood pressure worsening	M	1986	37.3	SCN	Yes
35.86	34%, AC 36% AFI UA N	PE symptoms, pathology abnormal		2180	9.2	SCN	–

**AC**: Abdominal Circumference, **AFI**: Amniotic Fluid Index, **BGL:** Blood Glucose Level, **BP:** Blood Pressure, **EDF**: Absent end-diastolic flow, **EFW**: Estimated Fetal Weight, **NICU**: Neonatal Intensive Care Unit, **SCN**: special care Nursery, **TTN**: Transient Tachypnea of the Newborn, **UA**: Umbilical Artery Doppler (**N**: Normal or **Absent).** Birthweight centiles were calculated using Australian birthweight percentiles adjusted for gestational age at delivery.^13^

## Discussion

This study is the first to describe the pharmacokinetics of sulfasalazine and sulfapyridine in pregnant individuals with preeclampsia. Sulfasalazine was well tolerated, with nausea being the most common adverse effects attributed to the drug. All neonates survived but most had complications, including small for gestational age (SGA) infants and neonatal intensive care unit (NICU) admissions. Furthermore, in contrast to *in vitro* studies where sulfasalazine treatment reduced sFlt-1 secretion,^4^^,^^6^ in our clinical cohort maternal sFlt-1 levels appeared to increase, particularly close to delivery. This rise may reflect the natural progression of preeclampsia rather than a direct drug effect. These findings suggest that the therapeutic potential of sulfasalazine may depend on timing of administration, whether used as prophylaxis earlier in gestation or initiated after diagnosis. Determining the optimal window of use will require evaluation in future randomised controlled trials. These outcomes are consistent with the expected clinical course of severe preterm preeclampsia and are unlikely attributable to sulfasalazine, which has been used safely in pregnancy for decades.^14^

Our study was designed to explore sulfasalazine and sulfapyridine pharmacokinetics in the mother and transfer to the fetus. We hypothesised that sulfasalazine pharmacokinetics would differ in this cohort due to gastrointestinal and hepatic alterations characteristic of preeclampsia.^1^ We find that the pharmacokinetic parameters for sulfasalazine in our preeclampsia cohort (3 g daily dose, median C_max_ 18.1 mg/L, T_max_ 2.0 h) were higher than sera of pregnant women on 2 g daily dose (C_max_ 4.6 ± 3.2 mg/mL).^15^^,^^16^ Similarly, sulfapyridine reached a delayed median T_max_ of 12 h. However, our comparison with this historical data is limited by in assay methodology, dosing regimens, sampling strategies, and populations studied. Therefore, the apparent difference in Cmax (e.g., 18.1 mg/L vs. ∼4.6 mg/L) cannot be interpreted as evidence of increased exposure in preeclampsia. Determining whether preeclampsia genuinely alters sulfasalazine PK will require contemporaneous non-PE pregnant controls analysed with the same methods.

A noteworthy finding from our study is the markedly high concentration of sulfapyridine observed in placental tissue, in some cases exceeding maternal and fetal plasma levels. While the maternal-to-cord plasma ratios for both sulfasalazine and sulfapyridine were approximately 1, suggesting similar distribution between maternal and fetal circulations, placental tissue concentrations, particularly for sulfapyridine, were higher.

Preeclampsia is known to affect hepatic enzyme activity, gastrointestinal motility, and protein binding capacity,^17^, ^18^, ^19^ all of which may influence drug bioavailability and clearance. Furthermore, sulfapyridine is primarily metabolised via N-acetylation, a process affected by genetic polymorphisms that greatly alter serum concentrations and elimination rates,^20^ which were not assessed in this study. Altered gut microbiota in pregnancy,^21^ particularly in those exposed to preeclampsia-associated inflammation,^1^^,^^22^ may have also impaired colonic bacterial conversion of sulfasalazine to sulfapyridine.

Placental transfer of both compounds was confirmed, with umbilical cord concentrations of sulfapyridine approaching or exceeding maternal concentrations in some cases. This is consistent with prior reports suggesting that sulfapyridine, being less protein-bound,^23^ crosses the placenta more readily, through passive diffusion, into cord sera than sulfasalazine.^24^^,^^25^ Given the high protein binding of sulfasalazine (98–99%), fetal and placental exposure is expected to be primarily determined by maternal free plasma concentrations. Placental drug measurements were therefore included to confirm transplacental exposure and to explore whether disease-related placental alterations in preeclampsia modify local drug distribution beyond maternal plasma levels, while recognising that maternal free and total plasma concentrations remain the most informative determinants of overall placental exposure.^26^

Sulfasalazine is generally safe in pregnancy, with no increased risk of congenital anomalies,^5^^,^^7^^,^^27^^,^^28^ though most data derive from participants with inflammatory bowel disease rather than hypertensive disorders. In our severe preeclampsia cohort, we also did not observe serious maternal or fetal harm with sulfasalazine treatment, though one participant was withdrawn due to worsening renal function. This was assessed as unlikely to be related to sulfasalazine, yet it highlights the importance of renal monitoring in future studies. Neonatal outcomes largely reflected the severity of preterm preeclampsia, including growth restriction and prematurity-related complications, with no congenital anomalies attributable to drug exposure. Nevertheless, ours is a small study that would need to be validated with a much larger cohort, and the clinical significance of fetal exposure remains uncertain, particularly if used chronically or earlier in gestation.

This study has several strengths. It is the first to explore sulfasalazine and sulfapyridine pharmacokinetics in the specific context of preeclampsia, a condition characterised by major physiological changes that can affect drug disposition. We also assessed both maternal and fetal exposure, providing data that are crucial for risk-benefit analysis in pregnancy drug trials. However, limitations include a small sample size, the absence of a control group of healthy pregnant individuals, and lack of genotyping for *NAT2* polymorphisms, which influences sulfasalazine metabolism and clearance.

Our findings raise important considerations for future research. Further studies should explore how preeclampsia-specific alterations in gut microbiota, hepatic function, and placental transport affect sulfasalazine and other colonic-activated prodrugs. Larger studies incorporating *NAT2* genotyping and longer sampling durations will help delineate pharmacokinetic subgroups and optimise dosing regimens. Given the measurable fetal exposure observed, mechanistic studies evaluating potential developmental or immunological effects of sulfasalazine *in utero* are also warranted.

In summary, our data describes sulfasalazine and sulfapyridine pharmacokinetics in pregnancies complicated by preeclampsia. The drug was well tolerated, and fetal exposure was confirmed. Clinically, our results support the cautious continued investigation of sulfasalazine as a potential therapy in maternal and fetal disorders, while underscoring the need to evaluate drug behaviour in the unique physiological context of preeclampsia. Our long-term goal is to determine whether sulfasalazine is a treatment for preeclampsia. A larger clinical trial examining efficacy and safety of the medication in participants with preterm preeclampsia can assist in assessing whether sulfasalazine could be a suitable treatment option in preeclampsia.

## Contributors

EK analysed part of the data and wrote the manuscript. EK and FB validated accessed and verified the data. VL and ZA developed and validated the LC-MS quantitation methods. ED and TK supervised the analytical method development, validation and sample analysis and reviewed the manuscript. VPFL conducted the non-compartmental analysis. CC edited the manuscript. AM served as Trial Manager, co-investigator, and member of the Trial Steering Committee (TSC). TJKL contributed as co-investigator and TSC member. ST was a principal investigator and TSC member. JMS was a principal investigator, coordinated recruitment at the Sunshine site, and served on the TSC. FB conceptualised the project, recruited participants, analysed the data, funded the project and wrote the manuscript. All authors reviewed the manuscript, providing input and approval of the final version.

## Data sharing statement

All individual participant data underlying the results reported in this article (after deidentification), along with a data dictionary, will be made available upon publication and for a minimum of seven years thereafter. Additional documents, including the study protocol, statistical analysis plan, and informed consent form, will also be available. Data can be accessed upon reasonable request by contacting the corresponding author at Fiona.brownfoot@unimelb.edu.au. Access will be granted to researchers whose proposed use of the data is approved by the corresponding author, following submission of a research proposal and completion of a data access agreement. Depending on the complexity of the request, data may be shared with or without investigator support.

## Declaration of interests

CC and ST provide consultancy services to Diamedica Therapeutics, Avilar Therapeutics and Beech Biopharma. CC and ST are on the scientific advisory board of Diamedica Therapeutics. FB is founder and part owner of Kali Health. ED provide consultancy services to Mediclinic Middle East Clinical Performance Committee. All other authors have nothing to declare.

## Appendix B Supplementary data

SIP trial protocol
