# Supplementary material for: Safety and pharmacokinetics of sulfasalazine and its metabolite sulfapyridine for treatment of preterm preeclampsia in Australia (SIP): an early phase, unblinded, single-arm, proof of concept clinical trial
Source: eClinicalMedicine. 2026 Feb 3;92:103779. doi: 10.1016/j.eclinm.2026.103779 (PMC12887773; doi:10.1016/j.eclinm.2026.103779)

Sulfasalazine Intervention for Preeclampsia (SIP) Study

Study Protocol:

Safety and pharmacokinetics of sulfasalazine used for the treatment of preterm preeclampsia: An early phase, unblinded, single-arm, proof of concept clinical trial

Sponsor	The University of Melbourne – Department of Obstetrics and Gynaecology
Investigational sites:	Mercy Hospital for Women (Mercy Health) and Sunshine Hospital (Western Health)
Funding Sources	This is an investigator initiated study.<br><br>Funding for the research will be provided in grants from the National Health and Medical Research Council; the Norman Beischer Medical Research Foundation; and the Society of Obstetric Medicine of Australia and New Zealand.
Principal Investigators	Dr Fiona Brownfoot and Professor Stephen Tong (Mercy Hospital for Women) and Assoc. Professor Joanne Said (Sunshine Hospital)
ANZCTR Number	ACTRN-1261-7000-2263-03
Mercy Health HREC Number	R16/65
TGA CTN Scheme Application ID	CT-2017-CTN-01723-1
Protocol Version Number and Date	Version 2 – 31st July, 2017

Amendments

Version No./Date	Amendment Type	Summary of Changes Made
V2 – 31/07/2017	Following ethical review and other non-substantial changes	• Gestation for inclusion maintained at 30+0 – 36+0 weeks<br>• Inclusion of brachial artery flow-mediated dilatation measurements (MHW participants only)<br>• Clarification of Doppler measurements to be performed (p. 24)<br>• Minor changes to pharmacokinetic sample collection details (p. 24, 30)<br>• Addition of *Section 6.1.7 – Antenatal Discharge* (p. 30)<br>• Creation and inclusion of a medication diary for discharged participants<br>• Minor typographical and layout corrections

CO-ORDINATING CENTRE TRIALS OFFICE:

University of Melbourne
Department of Obstetrics and Gynaecology

Level 4
Mercy Hospital for Women
163 Studley Road
Heidelberg
Victoria 3084

Telephone: +61 3 8458 4381
Fax: +61 3 8458 4380
Web:

www.mercyperinatal.com

http://medicine.unimelb.edu.au/school-structure/obstetrics-and-gynaecology

*Protocol authors

Co-ordinating Principal Investigator/ Site leader (Mercy Hospital for Women)	Principal Investigator	Trial Manager/ Associate Investigator
Dr Fiona Brownfoot* (MBBS, PhD, FRANZCOG)	Professor Stephen Tong (MBBS, PhD, FRANZCOG)	Ms Anna Middleton* (BAppSci(Nurs),PGradDip(Mid), MPH)
Maternal Fetal Medicine Fellow - Mercy Perinatal Unit<br>Research Fellow – University of Melbourne	Co-director – Mercy Perinatal<br>Head - Translational Obstetrics Group, University of Melbourne	Trial Coordinator - Mercy Perinatal;<br>Hon. Research Fellow - University of Melbourne
Level 4<br>Mercy Hospital for Women<br>163 Studley Road<br>Heidelberg, 3084	Level 4<br>Mercy Hospital for Women<br>163 Studley Road<br>Heidelberg, 3084	Level 4<br>Mercy Hospital for Women<br>163 Studley Road<br>Heidelberg, 3084
Tel: +61 3 8458 4381	Tel: +61 3 8458 4381	Tel: +61 3 8458 4381
Email: fiona.brownfoot@unimelb.edu.au	Email: stong@unimelb.edu.au	Email: amiddleton@mercy.com.au

**Principal Investigator/<br>Site leader (Sunshine Hospital)**<br><br>Associate Professor Joanne Said<br>(MBBS, Ph.D, FRANZCOG, CMFM, PGradDip(Epid))<br><br>Head of Maternal Fetal Medicine - Sunshine Hospital;<br>University of Melbourne<br><br>Tel: +61 8345 1333<br><br>Email: jsaid@unimelb.edu.au	**Associate Investigator**<br><br>Professor Susan Walker<br>(MBBS, MD, Dip RACOG, FRANZCOG, DDU, CMFM)<br><br>Co-director – Mercy Perinatal;<br>Head of Unit - University of Melbourne Department of Obstetrics & Gynaecology<br><br>Tel: +61 3 8458 4381<br><br>Email: spwalker@unimelb.edu.au	**Associate Investigator**<br><br>Dr Tu'uhevaha Kaitu'u-Lino<br>(BMedSci., Ph.D)<br><br>Senior Scientist – Translational Obstetrics Group,<br>University of Melbourne<br><br>Tel: +61 3 8458 4381<br><br>Email: t.klino@unimelb.edu.au
**Associate Investigator**<br><br>Ms Lee-Anne Lynch<br>(RN, RM, BHealthSci.(Nurs))<br><br>Clinical Research Midwife – Maternal Fetal Medicine Unit, Sunshine Hospital<br><br>Tel: +61 3 8395 8105<br><br>Email: lee-anne.lynch@wh.org.au	**Associate Investigator/<br>Trial Statistician**<br><br>Dr Richard Hiscock<br>(MBBS, FRANZCA, GradDip(Stat))<br><br>Anaesthetist – Mercy Hospital for Women<br><br>Tel: +61 8458 4393<br><br>Email: richardjhiscock@gmail.com	**Associate Investigator**<br><br>Mr James Dwyer<br>(BPharm, MPH, FSHP)<br><br>Director of Pharmacy – Mercy Hospitals Victoria Ltd.<br><br>Tel: +61 3 8458 4667<br><br>Email: jdwyer@mercy.com.au

Trial Committees

Trial Steering Committee

Professor Stephen Tong (Committee Chair) - University of Melbourne, Mercy Perinatal

Dr Fiona Brownfoot - University of Melbourne, Mercy Perinatal

Assoc. Professor Joanne Said - University of Melbourne, Sunshine Hospital

Ms Anna Middleton – University of Melbourne, Mercy Perinatal

Professor Susan Walker - University of Melbourne, Mercy Perinatal

Dr Tu'uhevaha Kaitu'u-Lino - University of Melbourne

Richard Hiscock – University of Melbourne, Mercy Anaesthetics

James Dwyer - University of Melbourne, Mercy Pharmacy

Data and Safety Monitoring Committee

Dr Stefan Kane (Committee Chair) – Royal Women's Hospital (Research; Obstetrics & Gynaecology)

Dr Jim Holberton – Mercy Hospital for Women (Neonatology; Paediatrics)

Dr Elizabeth McCarthy – Mercy Perinatal (Maternal Fetal Medicine; Obstetrics & Gynaecology)

Table of Contents

PROTOCOL APPROVAL (Cont.) 11
PROTOCOL APPROVAL (Cont.) 12
TRIAL SUMMARY 15
LAY SUMMARY 17
1 INTRODUCTION 18
1.1 BACKGROUND 18
Preeclampsia 18
Current management 18
Developing a medical treatment for preeclampsia 18
1.2 RATIONALE FOR THE STUDY 18
Potential new medical therapeutic option for preeclampsia – sulfasalazine 19
1.3 POTENTIAL RISKS TO PARTICIPANTS 19
1.4 POTENTIAL BENEFITS 20
1.4.1 Safe treatment for mothers; better outcomes for babies and families 20
1.4.2 Cost benefits 21
1.4.3 Reduction of maternal and fetal mortality in developing countries 21
2 STUDY OBJECTIVES 22
2.1 OBJECTIVES 22
2.1.1 Primary objectives 22
2.1.2 Secondary objectives 22
2.2 ENDPOINTS 23
2.2.1 Primary endpoint 23
2.2.2 Secondary endpoints 23
3 STUDY DESIGN 23
4 STUDY POPULATION 25
4.1 NUMBER OF PARTICIPANTS 25
4.2 INCLUSION CRITERIA 25
4.3 EXCLUSION CRITERIA 25
4.4 CO-ENROLMENT 25
5 PARTICIPANT SELECTION AND ENROLMENT 26
5.1 IDENTIFYING PARTICIPANTS 26
5.2 CONSENTING PARTICIPANTS 26

5.3 SCREENING FOR ELIGIBILITY 26
5.4 INELIGIBLE AND NON-RECRUITED PARTICIPANTS 26
5.5 RANDOMISATION 27
5.5.1 Treatment Allocation 27
5.5.2 Emergency Unblinding Procedures 27
5.6 WITHDRAWAL OF STUDY PARTICIPANTS 27
6 INVESTIGATIONAL MEDICINAL PRODUCT 28
6.1 STUDY DRUG 28
6.1.1 Study drug identification 28
6.1.2 Study drug manufacturer 28
6.1.3 ARTG authorization 28
6.1.4 Supply 29
6.1.5 Labelling and packaging 29
6.1.6 Storage 29
6.1.7 Manufacturer's Product Information/Investigators Brochure (IB) 30
6.2 DOSING REGIME 30
6.3 DOSAGE CHANGES 30
TABLE 1: Example study dose regime: 31
6.4 MEDICATION PRESCRIPTION AND PARTICIPANT COMPLIANCE 31
6.5 OVERDOSE 31
6.6 OTHER MEDICATIONS 32
6.6.1 Non-investigational medicinal products 32
6.6.2 Permitted medications 32
6.6.3 Other considerations: medications 32
7 STUDY ASSESSMENTS 32
7.1 SAFETY ASSESSMENTS 34
7.2 SUMMARY TABLE OF STUDY ASSESSMENTS 35
TABLE 2: Summary of study assessments 35
7.3 LONG TERM FOLLOW UP ASSESSMENTS 36
7.4 STORAGE AND ANALYSIS OF SAMPLES 36
8 DATA COLLECTION 37
9 STATISTICS AND DATA ANALYSIS 37
9.1 SAMPLE SIZE CALCULATION/RATIONALE 37

9.2 PROPOSED ANALYSES 37
9.2.1 Primary outcome analysis 38
9.2.2 Secondary outcome analyses 38
9.2.3 Missing data/sensitivity analyses 39
9.2.4 Subgroup analyses 39
9.2.5 Interim analysis 39
9.2.6 Final analysis 39
10 ADVERSE EVENTS 39
10.1 DEFINITIONS 39
10.2 IDENTIFYING AEs AND SAEs 40
10.3 RECORDING AEs AND SAEs 41
10.4 ASSESSMENT OF AEs AND SAEs 41
10.4.1 Assessment of seriousness 42
10.4.2 Assessment of causality 42
10.4.3 Assessment of expectedness 42
10.4.4 Assessment of severity 43
10.5 REPORTING OF SAEs/SADRs/SUSARs 43
10.6 REGULATORY REPORTING REQUIREMENTS 44
TABLE 3: TGA reporting requirements summary for serious events in clinical trials under CTN/CTX schemes: 44
10.7 FOLLOW UP PROCEDURES 45
11 PREGNANCY 45
12 TRIAL MANAGEMENT AND OVERSIGHT ARRANGEMENTS 45
12.1 TRIAL STEERING COMMITTEE 45
12.2 DATA AND SAFETY MONITORING COMMITTEE 46
12.3 INSPECTION OF RECORDS 46
12.4 RISK ASSESSMENT 47
12.5 STUDY MONITORING AND AUDIT 47
12.6 TRIAL MONITORING PLAN 47
13 GOOD CLINICAL PRACTICE 47
13.1 ETHICAL CONDUCT 48
13.2 REQUIRED APPROVALS AND CONDITIONS OF APPROVAL 48
13.3 REGULATORY COMPLIANCE 48

13.4 INVESTIGATOR RESPONSIBILITIES 48
13.4.1 Informed consent 48
13.4.2 Study site staff 49
13.4.3 Data recording 49
13.4.4 Investigator documentation 50
13.4.5 GCP training 50
13.4.6 Confidentiality 50
13.4.7 Intellectual Property 50
13.4.8 Data protection 51
14 STUDY CONDUCT RESPONSIBILITIES 51
14.1 PROTOCOL AMENDMENTS 51
14.2 PROTOCOL VIOLATIONS AND DEVIATIONS 52
14.3 SERIOUS BREACH REQUIREMENTS 52
14.4 STUDY RECORD RETENTION 52
14.5 END OF STUDY 52
14.6 CONTINUATION OF DRUG FOLLOWING THE END OF THE STUDY 53
14.7 INSURANCE AND INDEMNITY 53
15 REPORTING, PUBLICATIONS AND NOTIFICATION OF RESULTS 54
15.1 AUTHORSHIP POLICY 54
15.2 PUBLICATION 54
15.3 PEER REVIEW 55
REFERENCES 56
APPENDIX 1: Manufacturer's (Pfizer Australia) Product Information – Salazopyrin (sulfasalazine) 57
APPENDIX 2: Event reporting algorithm for sponsors 71

PROTOCOL APPROVAL

Sulfasalazine Intervention for Preeclampsia (SIP) Study

Safety and pharmacokinetics of sulfasalazine used for the treatment of preterm preeclampsia: An early phase, unblinded, single-arm, proof of concept clinical trial

Mercy Health HREC number – R16/65

TGA CTN Scheme ID number - CT-2017-CTN-01723-1

Australian and New Zealand Clinical Trials Registry (ANZCTR) number - ACTRN-1261-7000-2263-03

Co-ordinating Principal Investigator/Site Leader (MHW)

Fiona Brownfoot* Signature: Date: 31/07/2017

Principal Investigator

Stephen Tong Signature: Date: 31/7/2017

Trial Manager/Associate Researcher/Co-Investigator

Anna Middleton* Signature: Date: 31/07/2017

Associate Researcher/ Co-Investigator

Susan Walker Signature: Date:

Associate Researcher/ Co-Investigator

Tu'uhevaha Kaitu'u-Lino Signature: Date: 31/7/2017

***Protocol authors**

PROTOCOL APPROVAL (Cont.)

Sulfasalazine Intervention for Preeclampsia (SIP) Study

Safety and pharmacokinetics of sulfasalazine used for the treatment of preterm preeclampsia: An early phase, unblinded, single-arm, proof of concept clinical trial

Mercy Health HREC number – R16/65

TGA CTN Scheme ID number - CT-2017-CTN-01723-1

Australian and New Zealand Clinical Trials Registry (ANZCTR) number - ACTRN-1261-7000-2263-03

Site Leader (SH)/Associate Researcher/Co-Investigator

Joanne Said Signature: Date:

Associate Researcher/ Co-Investigator

Lee-anne Lynch Signature: Date:

Trial Statistician/Associate Researcher/Co-Investigator

Richard Hiscock Signature: Date:

Associate Researcher/ Co-Investigator

James Dwyer Signature: Date:

PROTOCOL APPROVAL (Cont.)

Sulfasalazine Intervention for Preeclampsia (SIP) Study

Safety and pharmacokinetics of sulfasalazine used for the treatment of preterm preeclampsia: An early phase, unblinded, single-arm, proof of concept clinical trial

Mercy Health HREC number – R16/65

TGA CTN Scheme ID number - CT-2017-CTN-01723-1

Australian and New Zealand Clinical Trials Registry (ANZCTR) number - ACTRN-1261-7000-2263-03

Data and Safety Monitoring Committee - Protocol Approval

Dr Stefan Kane (Chair)

Signature: Date: 7 September 2017

Dr Elizabeth McCarthy

Signature: Date:

Dr Jim Holberton

Signature: Date:

LIST OF ABBREVIATIONS

ADR	Adverse Drug Reaction
AE	Adverse Event
ANZCTR	Australian and New Zealand Clinical Trials Registry
ARTG	Australian Register of Therapeutic Goods
CRF	Case Report Form
CTN	Clinical Trial Notification
FBE	Full Blood Examination
GCP	Good Clinical Practice
HELLP	Haemolysis/Elevated Liver enzymes/Low Platelets (syndrome)
HREC	Human Research Ethics Committee
IB	Investigator's Brochure
ICH	International Conference on Harmonisation
IMP	Investigational Medicinal Product
IUGR	Intrauterine Growth Restriction
ISF	Investigator Site File
LFTs	Liver Function Tests
MHW	Mercy Hospital for Women
MSU	Mid-stream Urine
NHMRC	National Health and Medical Research Council
PE	Preeclampsia
PI	Principal Investigator
PICF	Patient Information and Consent
RWH	Royal Women's Hospital
SAE	Serious Adverse Event
SADR	Serious Adverse Drug Reaction

sENG	Soluble endoglin
sFlt-1	Soluble FMS-Like Tyrosine Kinase 1
SH	Sunshine Hospital
SL	Site Leader
SOP	Standard Operating Procedure
SUSAR	Suspected Unexpected Serious Adverse Reaction
TGA	Therapeutic Goods Administration
TMF	Trial Master File
TSC	Trial Steering Committee
UA Doppler	Umbilical Artery Doppler
UADR	Unexpected Adverse Drug Reaction
U&Es	Urea and Electrolytes

TRIAL SUMMARY

<table>
<tr><td>DESIGN:</td><td>An early (phase II), unblinded, single-arm, proof of concept clinical trial to assess the safety and pharmacokinetic profile of sulfasalazine as a treatment for preeclampsia in women who have a singleton, preterm pregnancy between 30+0 and 36+0 weeks gestation</td></tr>
<tr><td>SETTING:</td><td>Two public maternity hospitals in metropolitan Melbourne – Mercy Hospital for Women (MHW - Mercy Health); and Sunshine Hospital (SH - Western Health)</td></tr>
<tr><td>TARGET POPULATION:</td><td>We will recruit 20 women with singleton pregnancies (between 30+0 and 36+0 weeks gestation) admitted to hospital with a diagnosis of preeclampsia. Each participant will receive 3 grams of oral sulfasalazine (in divided doses) each day until delivery. This dose may be titrated down to 2 grams per day in case of side effects.

INCLUSION CRITERIA FOR THE STUDY:

Women who have/who are:
A singleton pregnancy between 30+0 and 36+0 weeks at the time of treatment initiation
Admitted to hospital with a diagnosis of preeclampsia as defined by the International Society for the Study of Hypertension in Pregnancy (1)
A pregnancy considered (by the treating obstetric unit) to be safe to continue for >12 hours after the time of recruitment
Absence of major fetal anomalies identified on morphology ultrasound
Aged 18-45 years at the time of recruitment
Capable of understanding all study-related information presented to them (written, oral and electronic)
Able to provide their written, informed consent to participate
EXCLUSION CRITERIA FOR THE STUDY:
A multiple pregnancy
Known major fetal malformation(s)
Contraindication(s) to sulfasalazine
Already taking sulfasalazine
Immunodeficiency disorder(s)
Unable to provide informed consent to participate</td></tr>
<tr><td>HEALTH TECHNOLOGIES</td><td>All recruited participants will receive 3 grams of oral sulfasalazine per day until delivery (see Appendix 1: Manufacturer's Product Information – Sulfasalazine)</td></tr>
</table>

ASSESSED:	
OUTCOME MEASURES:	**PRIMARY OUTCOMES:**<br><br>To establish the safety and pharmacokinetic profiles of oral sulfasalazine therapy in patients with preterm preeclampsia<br><br>**SECONDARY OUTCOMES:**<br><br>To determine the effects of oral sulfasalazine therapy on the clinical outcomes and biochemical markers of preeclampsia disease progression in preterm pregnancies
ANALYSIS:	• As there is no comparison arm, clinical variables and the clinical course will be presented as descriptive statistics<br>• There is no power calculation to perform<br>• Maternal and neonatal continuous variables will be statistically analysed using a t-test (parametric) or a Mann-Whitney test (non-parametric), as appropriate<br>• Categorical variables will be statistically analysed using a chi-squared test or Fisher's exact test, as appropriate<br>• Biomarker concentrations will be assessed as continuous variables<br>• Steady state sulfasalazine pharmacokinetic parameters will be estimated using standard non-compartmental techniques and normalized using actual body weights<br>• Statistical analysis will be performed using *GraphPad Prism 6* (GraphPad Software, La Jolla, CA)<br>• A p value of <0.05 will be considered statistically significant.
SAMPLE SIZE:	The researchers plan to recruit 20 women with preterm preeclampsia over a 2 year period, or until recruitment has been exhausted. It is anticipated that 15 participants will be recruited at Mercy Hospital for Women (MHW) and 5 will be recruited at Sunshine Hospital (SH).

LAY SUMMARY

Preeclampsia is a common, serious complication of pregnancy and is one of the leading causes of death, illness and disability in expectant mothers and their babies. Currently, there is no medical treatment for preeclampsia - the only way to halt the progression of the disease (and its associated risks to both the pregnant mother and her unborn baby) is to end the pregnancy by delivering the baby and placenta. When this occurs at an early gestation, it then exposes the newborn to the potentially life-threatening dangers associated with prematurity. Preeclampsia can also progress quite rapidly during the delivery process, putting the mother's health and life at immediate risk. Therefore, a treatment that stabilises the disease enough to allow the safe prolongation of affected pregnancies would be a major advance in the clinical care of pregnant women and their babies.

Excitingly, we have discovered that sulfasalazine - a medication that is already known to be safe in pregnancy - can reverse some of the key features of preeclampsia in laboratory studies. The use of primary human pregnancy tissues from donated maternal blood, cord blood and placental samples (including samples from women with preterm preeclampsia) has resulted in two significant laboratory findings:

- Sulfasalazine reduces the amount of preeclampsia-causing *anti-blood vessel factors* released by the placenta
- Sulfasalazine reduces the features of *vascular dysfunction* that are specific to preeclampsia.

Given these promising early results - coupled with sulfasalazine's documented safety profile during pregnancy - we hope to progress this concept and eventually translate these laboratory findings into clinical care. We propose to begin this process with a *proof of concept* early phase clinical trial.

We will recruit 20 women with preterm preeclampsia (between 30+0 weeks to 36+0 weeks gestation) at the Mercy Hospital for Women and Sunshine Hospital, and administer 3 grams of oral sulfasalazine per day. We will assess the safety and pharmacokinetic profiles of sulfasalazine in these women, as well as monitor key clinical and biochemical features of preeclampsia.

If successful, at the completion of this study, we will have sufficient evidence to progress the investigation of sulfasalazine as a treatment for preeclampsia to a large-scale, randomized clinical trial. We hope that it may form the basis for a future medical treatment for preeclampsia and reduce the devastating burden of this common obstetric disease.

1 INTRODUCTION

1.1 BACKGROUND

Preeclampsia

Preeclampsia affects 5-8% pregnancies and is a leading cause of obstetric and perinatal morbidity and mortality *(2, 3)*. It is characterised by the development of maternal hypertension and multisystem organ injury at >20 weeks gestation, which can progress to intracerebral bleeding; liver rupture; pulmonary oedema; renal failure; eclamptic seizures; and death. The fetus is also at increased risk of intra-uterine growth restriction (IUGR) and stillbirth (*4-6*).

Current management

Currently, there is no medical treatment for preeclampsia, with delivery the only option to halt disease progression (*7*). When preterm delivery of a pregnancy is indicated due to severe preeclampsia, the dangers associated with a premature birth put the neonate at an alarming risk of illness, disability, or even death. Furthermore, the disease mechanism of preeclampsia can progress rapidly in the mother during the delivery process, resulting in worsening multisystem organ involvement. Preeclampsia is responsible for 15% of maternal deaths and is the leading cause of iatrogenic preterm deliveries and subsequent neonatal death and disability (*8*). It poses a significant emotional and financial burden to families and society, with an estimated global economic cost of USD$3 billion annually.

The risk of neonatal morbidity and mortality related to preterm deliveries reduces significantly with each additional completed day and week of a healthy pregnancy. A treatment that slows or halts the disease process of preeclampsia would therefore be a significant scientific and clinical breakthrough from both a maternal and fetal/neonatal perspective - it could reduce maternal multisystem illness, whilst also allowing for the safe prolongation of pregnancies to a more advanced gestation.

Developing a medical treatment for preeclampsia

Historically, the development of a therapeutic treatment for preeclampsia has perhaps been hampered by a lack of understanding of the disease pathophysiology[5]. Excitingly, within the last 12 years, our field has begun to unravel this mystery with the discovery of two placentally-derived proteins - *Soluble FMS-Like Tyrosine Kinase 1* (sFlt-1)(*9-11*) and *Soluble Endoglin* (sENG)(*12*). These are thought to play a central role in the development of preeclampsia. Their secretion by the placenta is upregulated by hypoxia, inflammation and oxidative stress. Upon entering the maternal circulation, they cause widespread endothelial and blood vessel dysfunction, culminating in maternal multisystem organ injury(*4*). Therefore, a safe medical treatment that reduces the placental secretion of sFlt-1 and sENG while also improving endothelial dysfunction may be a therapeutic strategy to stabilise preeclampsia.

1.2 RATIONALE FOR THE STUDY

Potential new medical therapeutic option for preeclampsia – sulfasalazine

Sulfasalazine is an anti-inflammatory medication with antioxidant properties that is currently indicated for use in the treatment of inflammatory bowel disease and rheumatoid arthritis. Importantly, sulfasalazine therapy is able to be safely continued throughout a pregnancy (classified as a 'category A' drug) (*13*). Given sulfasalazine's acceptability as a therapy during pregnancy, coupled with its known anti-inflammatory and anti-oxidant properties, we examined whether it might mitigate key features of the preeclampsia disease process *in vitro*.

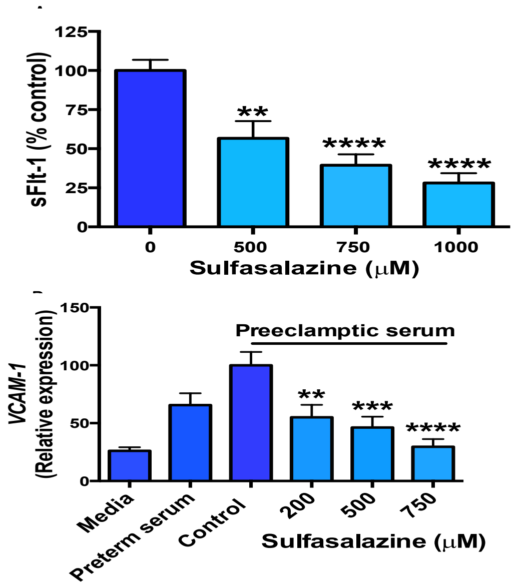

Figure 1: Sulfasalazine reduces A) sFlt-1 secretion from preeclamptic placenta and B) a marker of endothelial dysfunction VCAM-1 induced in endothelial cells with the addition of preeclamptic serum.

We have demonstrated that sulfasalazine reduces sFlt-1 and sENG secretion from human placenta, including from placental samples obtained from patients diagnosed with preterm preeclampsia (*Figure 1A*). Importantly, we have also shown that it can significantly improve vascular function by quenching markers of endothelial dysfunction (*Figure 1B*) and induce whole blood vessel dilation.

Given the promising *in vitro* data and sulfasalazine's established safety profile in pregnancy, we now wish to progress this concept to the clinic. We hope to recruit 20 participants with preterm preeclampsia and treat them with 3 g of oral sulfasalazine per day. The primary aims of this study are to:

- Assess the pharmacokinetic profile of sulfasalazine administered to pregnant women with preterm preeclampsia
- Gain further information on the safety profile of sulfasalazine therapy administered to women with preterm preeclampsia

Secondly, we will assess key clinical and biochemical features of preterm preeclampsia in women taking sulfasalazine. If effective, this will provide the first evidence that sulfasalazine may be a treatment for preeclampsia in humans. It will form the basis for a larger randomized controlled clinical trial aiming to establish sulfasalazine as a medical treatment for preeclampsia.

1.3 POTENTIAL RISKS TO PARTICIPANTS

We expect the proposed treatment will be safe and well tolerated. Sulfasalazine is classified as a 'category A' drug in pregnancy - it has historically been taken by large numbers of pregnant and child-bearing-aged women; and is not known to cause any increase in the frequency of direct harm, indirect harm, or anomalies in the developing fetus in either humans or animals (*13*). Importantly,

sulfasalazine therapy is currently continued as a treatment option for pregnant women who have concomitant rheumatoid arthritis or inflammatory bowel disease.

Possible fetal risks:

Not known to cause anomalies in/harm to the developing fetus (*13*).

Possible maternal risks:

Patients with preterm preeclampsia may experience discomforts or inconveniences relating to their admission to hospital, clinical examinations and blood tests, regardless of whether or not they decide to participate in the study. However, patients recruited to the study would undergo additional examinations and pathology tests that are supplementary to their routine clinical care, and as such, may experience an increase in these discomforts and inconveniences. These additional study requirements will be outlined in detail to all potential participants during the informed consent process.

Sulfasalazine is generally well-tolerated. Side effects relating to the medication can be either dose-related or idiosyncratic.

Dose-related side effects include gastrointestinal side effects (nausea, vomiting and diarrhoea), central nervous system (headache) and hematologic toxicities (including leukopenia, haemolytic anaemia and megaloblastic anaemia). Dose-related symptoms are generally experienced when taking sulfasalazine in larger doses (>4 grams per day) than we propose to administer in this study. Our participants will receive a maximum daily dose of 3 grams of sulfasalazine and will be monitored for dose-related side effects every 1-3 days by a member of the research team.

Idiosyncratic side effects of sulfasalazine are rare. They generally occur in the first 3 months of treatment, and symptoms may include skin rash, hepatitis, pancreatitis, pneumonitis, agranulocytosis and aplastic anaemia. These effects are usually dose-dependent. They are rarely seen in patients on low dose sulfasalazine, similar to the levels we propose to use for this study. Importantly, these side effects are generally transient and recovery is usually seen 1-2 weeks after ceasing the medication. Study participants will be monitored for the occurrence of idiosyncratic side effects in relation to taking sulfasalazine. However, as our study population will be made up of young, generally healthy women and we propose to use low doses of sulfasalazine therapy for a few weeks at most, we believe they would be at very low risk of developing these complications in relation to this study.

1.4 POTENTIAL BENEFITS

1.4.1 Safe treatment for mothers; better outcomes for babies and families

There are some potential benefits for participants related to their participation in the study. By offering a possible medical treatment for preeclampsia, we hope to quench the disease process using a well-tolerated medication with an established low-risk harm profile. Potential maternal benefits include disease stabilisation and a reduction in multisystem organ involvement (including

vessel; hepatic; renal; blood; and neurological disease), injury and failure. This could allow for the safe prolongation of some pregnancies to more advanced gestations. This would reduce premature delivery and the associated complications which include cerebral palsy, respiratory distress syndrome, chronic lung disease, retinopathy of prematurity, necrotising enterocolitis and other common morbidities of the preterm or growth-restricted neonate. Broadly, this treatment has the potential to reduce the short- and long-term social, practical, emotional and financial strains placed on women and families who experience preterm preeclampsia.

1.4.2 Cost benefits

Preeclampsia has an estimated global economic cost of USD$3 billion annually (*14*), with the impact borne by individuals, families, businesses, and health and welfare systems. Given that women are hospitalised with preeclampsia will be unable to perform their normal paid and unpaid occupations and carer responsibilities for a time, there can be a significant loss in productivity associated with the disease. A loss in household earning capacity (which often subsequently leads to an increase in reliance on government support) can remain throughout parents' working lives if they have a baby born with severe long-term sequelae, as well as possibly impacting on the child's ability to work and live independently once they reach adulthood.

There can be large healthcare costs associated with the inpatient treatment of women who develop preterm preeclampsia – this strain on the health care system can become even more substantial if the pregnancies result in the premature births of babies who then require a significant period of intensive hospitalisation or have long-term, complex health care needs. A medical treatment for preeclampsia could reduce this financial and social burden by reducing iatrogenic prematurity resulting in improved long-term health of the child.

1.4.3 Reduction of maternal and fetal mortality in developing countries

Although the burden of disease and economic costs associated with preeclampsia is significant in Australia, it is dwarfed by the impact endured by developing countries. Along with sepsis and haemorrhage, preeclampsia is one of the leading global causes of maternal and infant mortality, particularly in low-income settings (*15*). It is estimated that:

- There are approximately 830 maternal deaths globally every day from preventable causes in pregnancy and childbirth
- Approximately 99% of maternal deaths occur in developing countries
- Up to 25% of maternal deaths in some global regions are directly attributable to hypertensive disorders of pregnancy
- There are far greater numbers of fetal and neonatal deaths resulting from preeclampsia. (*16, 17*)

It is an important aspect of publically-funded research for the investigators to consider how any positive findings might be equitably translated for use into different health care contexts, such as in

rural and remote communities, developing countries and other resource-poor settings. Sulfasalazine is a medication already known to have a low-risk harm profile in pregnancy; is generally well-tolerated; is inexpensive; readily available; and vitally, for resource-poor or remote settings, does not require refrigeration to maintain its molecular stability. If sulfasalazine can be established as a safe and effective treatment for preeclampsia, it could be realistically translated into use in isolated communities within Australia, as well as being potentially appropriate for use in developing countries. This could potentially trigger an exponential improvement in the current global perinatal mortality figures, which are particularly poor in isolated communities and developing countries.

2 STUDY OBJECTIVES

An early (phase II) clinical trial assessing the safety and pharmacokinetic profile of sulfasalazine in patients with preeclampsia is required to assess the suitability of sulfasalazine as a potential medical treatment for preeclampsia.

2.1 OBJECTIVES

2.1.1 Primary objectives

The primary objective of this study is to establish the safety and pharmacokinetic profile of sulfasalazine in patients with preterm preeclampsia. In particular, we will be examining:

- Any adverse effects in the treated mother (safety data)
- Fetal/neonatal mortality and morbidity outcomes of participants' babies; including neurological, respiratory, eye and gastrointestinal disease (safety data)
- Maternal plasma and urine levels of circulating and excreted sulfasalazine following dose administration (pharmacokinetic data)
- Maternal blood, cord blood and placental levels of sulfasalazine following delivery (pharmacokinetic data)

2.1.2 Secondary objectives

To determine the effects of sulfasalazine on the clinical outcomes and biochemical markers of preeclampsia disease progression in preterm pregnancies by assessing for:

- Length of gestation prolongation (when compared with a contemporaneous cohort)
- Occurrence of clinical symptoms of preeclampsia
- Biochemical markers of preeclampsia disease; including but not limited to full blood count, Liver Function Tests (LFTs), renal function, proteinuria and haematological parameters
- Serum biomarkers of preeclampsia including but not limited to sFlt-1, sENG, Placental growth factor, endothelin 1, cellular adhesion molecules and cytokines.

2.2 ENDPOINTS

2.2.1 Primary endpoint

Delivery of the pregnancy.

2.2.2 Secondary endpoints

Laboratory endpoints/measures of interest:

- Serum biomarkers of preeclampsia (sFlt-1, sENG, placental growth factor, V-CAM, I-CAM and Endothelin; and 10 cytokine bioplex assay) during disease progression
- Biochemical markers of preeclampsia during disease progression

Clinical endpoints/measures of interest:

- Adverse Events (AEs) in mother and/or baby
- Time (days) to delivery of pregnancy after initial sulfasalazine dose
- Change in maternal systolic and diastolic blood pressure (mmHg)
- Change in maternal proteinuria (g/mmol)
- Emergence of/change in other clinical symptoms of preeclampsia
- Maternal development of Haemolysis/Elevated Liver enzymes/Low Platelets (HELLP) syndrome
- Neonatal morbidity/mortality outcomes
- Acceptability of treatment and side effects to participants

3 STUDY DESIGN

This study is an early (phase II), unblinded, single-arm, proof of concept clinical intervention trial aiming to assess the safety and pharmacokinetic profile of sulfasalazine as a treatment for preterm preeclampsia.

20 participants with singleton pregnancies between 30+0 and 36+0 weeks gestation will be recruited over a two year period. Recruitment will occur at the Mercy Hospital for Women (15 participants) and Sunshine Hospital (5 participants) following a patient's antenatal admission to hospital with a diagnosis of proteinuric preeclampsia, as per the Society for the Study of Hypertension in pregnancy guidelines(*1*).

All recruited participants will receive 3 grams of sulfasalazine upon enrolment in the study. The following pathology tests will be collected in relation to the study:

Once the participant has been taking sulfasalazine for >30 hours:

- 6 x blood samples (≥5 ml per sample collected in pink-topped plasma EDTA tubes) to assess maternal plasma levels and pharmacokinetics of sulfasalazine will be collected from the patient's dedicated intravenous cannula during one 12 hour period
- 3 x Mid-stream urine (MSU) samples (≥5 ml per sample) to assess for maternal sulfasalazine excretion will be collected in one 12 hour period.

<u>During remainder of pregnancy:</u>

- When blood tests are collected as part of clinical care, an additional sample (≥5 ml per sample collected in pink-topped plasma EDTA tube) will be collected to assess maternal serum biomarkers of preeclampsia including:
    - Anti-angiogenic factors associated with preeclampsia (sFlt-1 and sENG)
    - Biomarkers of endothelial injury (V-CAM, I-CAM and Endothelin)
    - 10 cytokine bioplex assay

<u>Postnatally:</u>

- Maternal blood (1 x pink-topped plasma EDTA tube), umbilical cord blood (1 x pink-topped plasma EDTA tube) and placental samples (6 x 30 g tissue samples) will be collected at delivery to assess for circulating sulfasalazine levels in both mother and baby

The hospitals' local Standard Operating Procedures (SOP) of managing preterm preeclampsia (including blood tests ≥3 times per week; twice weekly growth and wellbeing ultrasounds; *umbilical artery (UA) Doppler* and CTG monitoring 3 times per week) - or management as otherwise ordered by the treating obstetric unit – will be followed.

Participants will be visited every 1-3 days from recruitment until delivery (while they remain inpatients) by a member of the research team to monitor for AEs or possible side effects of sulfasalazine, and to ensure the participant's ongoing willingness to participate in the trial. Participants who are discharged home prior to the birth of their baby will be reviewed by a researcher each time they attend their hospital for an outpatient review until they are readmitted or their baby is born. A member of the research team will be on call to attend or for advice relating to concerns raised by the participants or the clinicians providing their direct care.

Biochemical markers of preeclampsia disease (such as LFTs and proteinuria) will be monitored as a part of standard clinical care. The researchers will also have access to these routine pathology results – this data forms some of the secondary outcomes of the study.

The timing of delivery and any deviations from routine clinical care will be determined by the treating clinician or obstetric unit – not by a member of the research team. In the case where a member of the research team is also acting as the treating clinician, these decisions will be made in consultation with other members of the treating obstetric unit, with the best interests of mother and baby as the paramount consideration.

The medical histories of babies born to participants (including any pathology results from tests ordered by the treating clinicians) will be reviewed postnatally to assess for neonatal outcomes and AEs. A ‘closing out’ visit on the postnatal ward (or phone call following maternal discharge) will be made following the delivery of each participant’s baby.

4 STUDY POPULATION

4.1 NUMBER OF PARTICIPANTS

The researchers plan to recruit 20 women with preterm preeclampsia between 30+0 and 36+0 weeks gestation, over a period of 2 years. The researchers believe that this sample size is justified, as outlined in *Section 9.1* of this document.

4.2 INCLUSION CRITERIA

Women who have/who are:

- A singleton pregnancy between 30+0 and 36+0 weeks at the time of treatment initiation
- Admitted as hospital inpatients with a diagnosis of preeclampsia as defined by the International Society for the Study of Hypertension in pregnancy guidelines(*1*)
- A pregnancy considered (by the treating obstetric unit) to be safe to continue for >12 hours after the time of recruitment
- Absence of major fetal anomalies identified on morphology ultrasound
- Aged 18-45 years at the time of recruitment
- Capable of understanding all study-related information presented to them (written, oral and electronic)
- Able to provide their written, informed consent to participate

4.3 EXCLUSION CRITERIA

Women who have/who are:

- A multiple pregnancy
- Known major fetal malformation(s)
- Contraindication(s) to sulfasalazine
- Already taking sulfasalazine
- Immunodeficiency disorder(s)
- Unable to provide informed consent to participate

4.4 CO-ENROLMENT

Participants will not be eligible to take part in any other intervention trials for the duration of their enrolment in this study. Participants may elect to take part in other observational research (e.g. tissue bank donations, questionnaire studies, etc.) as they wish.

5 PARTICIPANT SELECTION AND ENROLMENT

5.1 IDENTIFYING PARTICIPANTS

Women with a singleton pregnancy between 30+0 and 36+0 weeks gestation who are diagnosed and admitted to hospital with preeclampsia will be identified by the research team from hospital admission data sources or referred by clinical staff (in Birthing Suites, Emergency, Antenatal Clinic and the Fetal Monitoring/Pregnancy Day Stay Units) to the research team.

5.2 CONSENTING PARTICIPANTS

Eligible participants will be identified by their clinical care team and referred to the research team or identified by the researchers from hospital admission data. A member of the research team will approach potential participants and provide them with a verbal explanation of the study, as well as a written or electronic copy of the *Patient Information and Consent Form* (PICF). All eligible women will have the opportunity to discuss and ask questions about the study with a member of the research team who is also an obstetrician or obstetric trainee prior to consenting. Informed, written consent will only be collected once the patient has had adequate time to read and reflect upon the study information and had all questions answered to her satisfaction. Recruited participants will retain their copy of the PICF and be given a copy of their signed consent form for their personal records.

5.3 SCREENING FOR ELIGIBILITY

To be eligible for the study, a woman must have a singleton pregnancy between 30+0 and 36+0 weeks gestation, with a pending or current inpatient admission for a diagnosis of preeclampsia according to the Society for the Study of Hypertension in pregnancy guideline(*1*). Additionally, the woman must be aged 18-45 years; capable of understanding the study information presented to her; have the ability to provide written consent; considered by the treating obstetric team to be safe to continue the pregnancy for >12 hours; and have a normal fetal morphology scan/aneuploidy testing result(s) (if performed) for this pregnancy. These parameters will be applied to determine eligibility for the study for all women referred to or identified by the research team. Eligibility (or non-eligibility) will be documented by a member of the research team in the medical history of all patients screened for inclusion in the study.

5.4 INELIGIBLE AND NON-RECRUITED PARTICIPANTS

No further information will be collected on women once they are deemed to be ineligible to participate in the study, or once they decline or withdraw consent for their inclusion. Anonymised information will be collected on a password protected, online screening log to capture all patients assessed for their eligibility, whether or not they are recruited. Those women who are not recruited

into the study or who withdraw their consent to participate will continue with standard clinical care for the treatment of their preeclampsia, as per their treating hospital's SOP and treating obstetric unit instruction.

5.5 RANDOMISATION

Not applicable – this is a single arm, unblinded study.

5.5.1 Treatment Allocation

Not applicable.

5.5.2 Emergency Unblinding Procedures

Not applicable.

5.6 WITHDRAWAL OF STUDY PARTICIPANTS

Participation in the study is completely voluntary. Participants will be made aware of their ongoing right to discontinue taking sulfasalazine or to withdraw entirely from the study at any time, and for any reason. The research team also retains the right to discontinue a participant from taking sulfasalazine and initiating their withdrawal from the study at any time, if it is deemed by a Principal Investigator or Site Leader to be in the patient's and/or her baby's best interest(s).

If a participant is withdrawn from the study by a researcher due to a *Serious Adverse Event* (SAE), the relevant hospital Site Leader will arrange for medical follow-up visits or other contact with the treating hospital until the event has been resolved or stabilised. Any data collected from the participant up until their removal from the study will still be used in statistical analyses, unless this is specifically refused by the participant.

If a participant withdraws their consent to the study at any point, no further data will be collected about them by the research team. However, permission will be sought to continue using anonymised data and research samples collected from the participant up until the point of withdrawal. The research team will attempt to identify with the participant the reason/s for their withdrawal, in case a study protocol amendment and/or medical follow-up visits are warranted.

The research team will make reasonable attempts (i.e. attempting to make contact on $\geq$3 occasions, using more than one mode of communication) to locate a participant before they are deemed to be 'lost to follow-up'. If a participant is deemed to be lost to follow-up, but has not indicated to the research team that they wish to be withdrawn from the study, their anonymised data and any collected samples will still be used in the final analysis.

6 INVESTIGATIONAL MEDICINAL PRODUCT

6.1 STUDY DRUG

Generic: Sulfasalazine

Traded as: Salazopyrin EN (Pfizer Australia Pty. Ltd.)

6.1.1 Study drug identification

Salazopyrin EN (sulfasalazine) enteric coated – *Australian Register of Therapeutic Goods* (ARTG) ID 14485 – supplied in bottles of 100 x 500 mg tablets.

Physical description: Elliptical convex, yellow-orange, enteric coated tablets marked with *KPh* on one side; *102* on the reverse.

6.1.2 Study drug manufacturer

Pfizer Health AB

Bjorkgarten 30

S-75182

Uppsala

Sweden

Tel: +46 1522 7300

Fax: +46 8618 8607

6.1.3 ARTG authorization

ARTG ID 14485

Drug sponsor:

Pfizer Australia Pty. Ltd.

38-42 Wharf Road

West Rhyde

NSW, 2114

Tel: 1800 675 229 (Medical information and adverse event reporting)

+61 2 9850 3333 (General enquiries)

Web: www.pfizer.com.au

6.1.4 Supply

The on-site hospital pharmacies will supply sulfasalazine with project funds. Upon receipt of a medication chart order/reorder of sulfasalazine for a study participant, pharmacy will dispense a suitable supply of the medication to the ward (if the participant is an inpatient) or to the participant (if an outpatient). The supply should be sufficient to last approximately five days (30 tablets). A small supply of sulfasalazine will be kept in the after-hours medication cupboard at each investigational site, in case of a participant commencing participation in the trial outside of the hospital pharmacists' normal working hours.

6.1.5 Labelling and packaging

Sulfasalazine will be supplied to the admitting ward for each individual participant by the hospital pharmacies in accordance with local SOPs for prescription study medication labelling and packaging. As this is not a blinded study, sulfasalazine can still be identified by its generic name, trade name and dose on the packaging/label.

6.1.6 Storage

Sulfasalazine will be securely stored in the hospital pharmacies, and will be dispensed to the admitting wards for specific trial participants or to the participant themselves upon receipt of an inpatient medication chart or prescription. Tablets will be kept secure on the wards in locked medication drawers or rooms, as per the local hospital SOP for the safe storage and management of prescription medications. While tablets remain in the hospital, they will be kept at room temperature (<25 °C), as per the manufacturer's guidelines (see *Appendix 1*). Upon delivery of a participant's baby or at their withdrawal from the study, unused tablets will be returned to the hospital pharmacy.

6.1.7 Antenatal discharge

The majority of women enrolled in the study will likely remain as inpatients until delivery of their pregnancy. In the event that a participant is discharged home for a period of time prior to delivery, she may continue to participate in the study - a take home supply of the drug (with detailed dosage instructions) and medication diary will be arranged prior to her discharge. The medication diary will outline emergency contact details in the event of a participant experiencing an adverse event outside of the hospital. A member of the research team will meet with the discharged participant during her subsequent outpatient visits to the hospital to ensure her ongoing wellbeing while taking the study drug; assess study protocol compliance by reviewing the participant's medication diary and count the number of remaining tablets; and to arrange a further supply of sulfasalazine from pharmacy, as needed. Discharged participants will be instructed to store their sulfasalazine at <25 °C, as per the manufacturer's instructions.

6.1.8 Manufacturer's Product Information/Investigators Brochure (IB)

The manufacturer's Product Information for sulfasalazine (traded as Salazopyrin EN) is given in *Appendix 1*.

6.2 DOSING REGIME

Trial subjects will receive a daily total of 3 grams of sulfasalazine, divided into 2 equal doses of 1.5 grams each, to be administered post-breakfast and post-dinner. Salazopyrin EN tablets should be taken whole, following a meal, as per the manufacturer's guidelines. The doses should be as evenly spaced as possible.

6.3 DOSAGE CHANGES

In the event that a participant experiences mild to moderate gastrointestinal side effects from taking sulfasalazine, but still wishes to participate in the trial, the dosage regime would be titrated down to a total of 2 grams per day in an attempt to mitigate the severity of the symptoms. If these symptoms resolve, the Site Leader may titrate the participant's dose back up to 3 grams daily according to their clinical judgement. Any participants who experience severe side effects associated with sulfasalazine therapy will be withdrawn from the study and revert to standard clinical management of preeclampsia. See *Section 10.4.4* for definitions of event severity.

TABLE 1: Example study dose regime:

	**Post-breakfast**	**Post-dinner**	**Total daily dose**
**Routine study sulfasalazine regime**	1.5 gram (3 x 500mg tablets)	1.5 gram (3 x 500mg tablets)	3 grams (6 x 500mg tablets)
**Altered sulfasalazine regime if side effects**	1 gram (2 x 500mg tablets)	1 gram (2 x 500mg tablets)	2 grams (4 x 500mg tablets)

6.4 MEDICATION PRESCRIPTION AND PARTICIPANT COMPLIANCE

All participants will begin the study as admitted inpatients, with sulfasalazine ordered as regular doses (1.5 grams oral sulfasalazine, twice daily, after food) on their hospital medication chart. The prescribing doctor should denote *'PE sulfasalazine trial'* as the indication on the medication chart, and tick the '*Slow Release'* box.

The timing or refusal of sulfasalazine doses will be documented on the participant's medication chart by the treating midwife or other clinician, as per the hospital's local SOP for medication administration and documentation. A member of the research team will review the patient's medication chart to assess for compliance or deviations from the study protocol of sulfasalazine at each study-related visit.

Any unused sulfasalazine from the supply dispensed for a particular participant will be returned to the local hospital pharmacy at the conclusion of the patient's study participation (as outlined in *Section 6.1.6*).

In the case of a participant who is discharged home prior to delivery, the researchers will rely on verbal reports and medication diary entries of medication regime compliance, confirmed with pill counts of remaining tablets at each outpatient visit attended by the participant.

6.5 OVERDOSE

According to the Manufacturer's information (*Appendix 1*), sulfasalazine has a low per oral toxicity, except in the case of a hypersensitivity reaction. Similar to other sulphonamide drugs, the common signs and symptoms of a hypersensitivity reaction to sulfasalazine include gastrointestinal symptoms (nausea, vomiting and diarrhoea), haematuria, crystaluria or anuria. Central nervous system symptoms such as dizziness or convulsions may occur in more advanced cases, although it is not anticipated that participants will be on a high enough dose of sulfasalazine for long enough for these advanced symptoms to develop in relation to the medication (see *Appendix 1*). There is no specific

antidote for sulfasalazine overdose – any reactions should be treated symptomatically and supportively. Withdrawal of a participant from the trial following a sensitivity reaction will be considered and discussed between the research team, the participant and the participant's treating obstetric unit, and will depend on the nature and severity of the reaction. The *Victorian Poisons Information Centre* can be contacted any time on 13 11 26 for advice in the event of a suspected overdose.

6.6 OTHER MEDICATIONS

6.6.1 Non-investigational medicinal products

There is no current standard medical treatment option for the management of preeclampsia, however antihypertensive and other routine medication(s) (e.g. labetalol, nifedipine, methyldopa, hydralazine, magnesium sulphate, folic acid, iron supplementation etc.) will be concurrently ordered for the management of this patient cohort.

6.6.2 Permitted medications

As outlined in the manufacturer's Product Information *(Appendix 1)*.

6.6.3 Other considerations: medications

For full information, see the manufacturer's Product Information *(Appendix 1)*. Consideration should be taken with the co-administration of sulfasalazine with the following drugs:

- *Antacids* – Known to decrease the absorption of sulphonamides
- *Penicillins* – Sulphonamides may inhibit absorption and effectiveness of penicillins
- *Local anaesthetics derived from Para-amniobenzoic acid* – May antagonise sulphonamide activity
- *Digoxin* – Concomitant administration may cause reduced absorption of digoxin to sub therapeutic levels
- *Folic acid* – Folate deficiency may occur when taking sulfasalazine – co-administration of 1-2 grams daily of folic acid during pregnancy is recommended
- *Ferrous sulphate* – May inhibit the absorption and effectiveness of sulfasalazine
- *Azathioprine* – Bone marrow suppression and leucopenia have been reported with co-administration. Appropriate monitoring should occur with any co-administration.

7 STUDY ASSESSMENTS

<u>STUDY ASSESSMENTS PRIOR TO TREATMENT</u>

As part of routine clinical care, examinations and pathology tests will have been carried out prior to a participant's screening for recruitment. The results of these tests and examinations help to establish a patient's suitability for the study and give additional details that may be pertinent to the study analysis for stratification purposes. The following test and examination results will be transcribed as appropriate into the *Case Report Form* (CRF), following a participant consenting for inclusion in the study:

- Gravida and parity
- Estimated due date
- Height, weight and BMI at booking and at recruitment
- Blood pressure readings throughout pregnancy
- Medical progress notes tracking the history of the evolution of preeclampsia
- Pathology tests (including but not limited to *Full Blood Examination* (FBE); *Urea and Electrolytes* (U&Es); LFTs; Uric Acid; Oral Glucose Tolerance Test; coagulation profile; UA Dopplers; growth and wellbeing ultrasounds; etc.)
- Urine spot protein/creatinine ratio; and/or 24-hour urine collection
- First and/or second trimester dating ultrasound
- Aneuploidy screening tests
- Morphology ultrasound

STUDY ASSESSMENTS - DAY OF INITIAL TREATMENT DOSE

- Written, informed consent obtained
- Medical history review
- Discussion with participant's treating doctors and midwives
- Eligibility confirmed by senior member of the research team
- CRFs completed
- Sulfasalazine prescribed on medication chart by an obstetric doctor
- Assess for *Adverse Drug Reaction* (ADR) 30-180 minutes following administration of the initial dose
- At MHW only – Brachial artery flow-mediated dilatation measurements performed before first dose of sulfasalazine and after steady state has been achieved > 30 hours post commencing sulfasalazine.

STUDY ASSESSMENTS - AFTER 30 HOURS OF SULFASALAZINE TREATMENT

- Insertion of a dedicated intravenous cannula
- Six blood (≥5 ml collected in pink-topped plasma EDTA tubes each time) tests over one 12 hour period (via the cannula)
- 3 x MSU samples (≥5 ml per sample, collected in yellow-topped sterile specimen pots) over one 12 hour period
- At MHW only – Repeated brachial artery flow-mediated dilatation measurements

After these specific samples and measurements are collected, study assessments will follow the pattern of normal clinical care as per local SOP for the treatment of preeclampsia. Study-specific blood samples (≥5 ml collected in pink-topped plasma EDTA tubes) to assess for maternal serum biomarkers of preeclampsia will be collected along with any clinically indicated blood tests, as dictated by the treating obstetric team (usually every 2-3 days).

STUDY ASSESSMENTS – POSTNATAL

- Collection of 1 x pink-topped plasma EDTA tube (maternal blood) at delivery
- Collection of 1 x pink-topped plasma EDTA tube (cord blood)
- Collection of 6 x 30g placental samples
- Postnatal closing out visit/phone call
- Maternal and neonatal history reviews

Other information required for study assessments (such as blood test results, growth and wellbeing ultrasounds, etc.) that form a normal part of clinical care will be transcribed into the CRF from patient files.

Clinical management may vary slightly between the two participating sites or between the different obstetric units therein. A member of the research team will visit each participant every 1-3 days (or more frequently, as requested by the direct clinical care team), communicate with their clinical carers and review the participant's medical history. The researcher will update the CRF detailing:

- Any AEs experienced by the participant
- Any changes in severity of the signs or symptoms of the preeclampsia disease process
- Any pathology tests undertaken since the last study visit
- Compliance with treatment regime
- Participant wellbeing
- The participant's stated willingness to continue with the study

7.1 SAFETY ASSESSMENTS

- Trial participants will have baseline observations performed prior to the administration of the 1st dose of sulfasalazine, and will have their wellbeing reassessed by a study Investigator within 3 hours of taking the 1st dose.
- Trial participants will be monitored for serum levels of sulfasalazine as part of the pharmacokinetic component of the trial. These levels are thought to be directly related to incidences of sulfasalazine-induced hypersensitivity reactions and toxicity (see *Section 6.5* and *Appendix 1*).

- As a part of the routine management for women with preterm preeclampsia, participants will undergo frequent, multimodal monitoring of their own condition, as well as that of their baby. This management and these results will be overseen by the participants' treating clinicians, with additional reviews performed by the research team during the collation of results into the CRF.
- All participants will be public hospital inpatients for at least the initial phase of their study involvement, with 24 hour midwifery, obstetric and critical care staff available in case of a serious medical event
- Following delivery of their baby and the cessation of the sulfasalazine therapy, each participant will undergo routine monitoring and follow-up of their preeclampsia to ensure its timely resolution

  Following any AE involving a participant, an obstetrician who is also a member of the research team will review the patient and the history of the AE to establish severity and causality of the event (see *Section 10*), determining whether or not the event was likely attributed to sulfasalazine. They will also arrange for any necessary follow up with the patient in an attempt to ensure that the AE is resolved satisfactorily.

7.2 SUMMARY TABLE OF STUDY ASSESSMENTS

TABLE 2: Summary of study assessments

	Prior to treatment commencement	1st day of treatment	After 30 hours of treatment	Each subsequent review	Day of delivery (or next weekday)	Closing out visit/phone call
**Consent**	✔	✔*	✔*	✔*	✔*	✔*
**Medical history review**	✔	✔	✔	✔	✔	✔
**Check results: Clinical pathology tests**	✔	✔	✔	✔	✔	✔
**Flow-mediated dilatation (MHW)**	✔		✔			
**Sulfasalazine levels**			✔**		✔***	
**Biomarkers of PE**	✔	✔	✔	✔	✔	
**Adverse events**		✔	✔	✔	✔	✔

**IMP regime compliance**		✔	✔	✔	✔	
**Neonatal history review**					✔	✔

*** = Verbally confirm ongoing consent**

**** = Maternal serum/urine levels taken following 30 hours of sulfasalazine therapy**

***** = Maternal blood, placental and cord blood levels**

7.3 LONG TERM FOLLOW UP ASSESSMENTS

At the time of application, there are no plans for long term follow up of the study cohort.

7.4 STORAGE AND ANALYSIS OF SAMPLES

The research team will organise for the collection, transport, short-term storage, processing, analysis and disposal of the following blood, urine and placental samples:

- 6 x blood samples collected during one 12 hour period ≥30 hours following the 1st dose of sulfasalazine to assess for maternal plasma levels and pharmacokinetic activity of sulfasalazine
- 3 x MSU samples collected during one 12 hour period following the commencement of sulfasalazine therapy dose to assess for maternal excretion of sulfasalazine and its metabolites
- Subsequent study blood samples collected concurrently with routine pathology to assess for maternal serum biomarkers of preeclampsia
- Maternal blood, cord blood and placental samples collected at delivery

Research samples for this study will be managed at the University of Melbourne Department of Obstetrics and Gynaecology laboratory at the Mercy Hospital for Women and the research laboratory at Sunshine Hospital. These samples will be handled and analysed by appropriately qualified members of the research team and laboratory staff in accordance with University of Melbourne laboratory SOPs. These samples will be disposed of as soon as they are processed and the required data has been extracted.

All other pathology samples will be sent for analysis to each participating site's contracted pathology provider. The research team will only access the results of these tests from patient files to be

transposed into the CRF. Responsibility for the ordering, collection, transport, analysis, storage, and disposal of these samples will remain with the clinical care team and the local pathology service providers (as appropriate).

8 DATA COLLECTION

The participants' medical records and pathology results are considered to be source data. Information will be transcribed or copied (with abridgments, where appropriate) into the CRF. Data will be collected and transcribed as it becomes available, at each point of contact that the research team has with the participant. It is anticipated that with a small sample size and multiple Co-Investigators/Associate Researchers, participant follow-up will be well-attended and missing data will be kept to a minimum. Where possible, data will be collected from patients regardless of their compliance with the treatment regime (see *Section 9.2.3*). Study protocol compliance will be commented on in the study analysis. The researchers will attempt to collect and report on reasons for any missing data or deviations from study medication regime.

9 STATISTICS AND DATA ANALYSIS

9.1 SAMPLE SIZE CALCULATION/RATIONALE

The researchers plan to recruit 20 women with preterm preeclampsia over a period of 2 years. The researchers believe that this sample size is justified as follows:

- Many 'first in man' studies will recruit 10 to 20 participants. Whilst sulfasalazine is already given to pregnant women for other treatment indications, it has not yet been administered to an exclusively preeclamptic cohort
- 20 is a modest number, however it will be sufficient to obtain pharmacokinetic information and give preliminary data about sulfasalazine's capacity to have a clinical or biochemical effect on the disease mechanisms of preeclampsia
- A recent case report published in the American Journal of Obstetrics and Gynaecology (*18*) examined the effect of pravastatin in a sample size of 20 pregnant patients. This study primarily assessed the pharmacokinetics of pravastatin in pregnancy and also observed the incidence of preeclampsia
- The researchers are targeting preterm preeclampsia, which is a rarer occurrence than term preeclampsia. 20 participants is a realistic number that can be recruited over 2 years, when accounting for those cases that will inevitably decline inclusion or will be otherwise excluded from participating.

9.2 PROPOSED ANALYSES

Statistical analysis for this study will be performed using *GraphPad Prism 6* (GraphPad Software, La Jolla, CA). Given the study design and objectives of this particular trial:

- There is no comparison arm, so clinical variables and the clinical course will be presented as descriptive statistics
- There is no power calculation to perform
- Maternal and neonatal continuous variables will be statistically analysed using a t-test (parametric) or a Mann-Whitney test (non-parametric), as appropriate
- Categorical variables will be statistically analysed using a chi-squared test or Fisher's exact test, as appropriate
- Biomarker concentrations will be assessed as continuous variables
- Steady state sulfasalazine pharmacokinetic parameters will be estimated using standard non-compartmental techniques and normalized using actual body weights
- A p value of <0.05 will be considered statistically significant.

9.2.1 Primary outcome analysis

- The primary objective of this study is to assess the safety of administering sulfasalazine in patients with preterm preeclampsia
- We will assess for adverse effects in the mother and the fetus/neonate
- We will assess mortality and morbidity outcomes - including neurological, respiratory, eye disorders and gastrointestinal disease - in the fetus/neonate
- We will also assess the pharmacokinetic profile of sulfasalazine which will involve taking maternal serum levels of sulfasalazine following administration; maternal urinary excretion of sulfasalazine following administration; and maternal serum levels, cord blood levels and placental levels of sulfasalazine at delivery
- Pharmacokinetic parameters include maximum concentration (C-max); time to maximum concentration (T-max); area under the concentration time curve (AUC); apparent oral clearance (CL/F); half-life; and renal clearance.

9.2.2 Secondary outcome analyses

- The secondary objective will be to determine the effects of sulfasalazine on clinical and biochemical markers of preeclampsia disease progression.
- In the mother, we will assess length of gestation prolongation compared to a contemporaneous cohort; occurrence of clinical symptoms; and the progression of biochemical aspects of disease (including liver function, renal function, proteinuria and haematological parameters).
- For participants recruited at MHW, we will measure brachial artery flow-mediated dilatation as a surrogate measure of maternal endothelial function
- Furthermore, we will assess biomarkers of preeclampsia (including sFlt-1 and sENG) in maternal blood samples from recruitment until delivery

9.2.3 Missing data/sensitivity analyses

Missing data will be presented descriptively.

9.2.4 Subgroup analyses

This study will not be powered to perform subgroup analyses.

9.2.5 Interim analysis

An interim analysis will not be conducted for this study.

9.2.6 Final analysis

A final analysis will be conducted by the trial Investigators.

10 ADVERSE EVENTS

The Manufacturer's Product Information (see *Appendix 1*) for Salazopyrin EN contains the full details of the known contraindications and side effects that have been reported following the administration.

Participants who are recruited to the trial will be instructed to inform their direct clinical care team or a member of the research team at any time during their participation if they develop symptoms, whether or not they think these may be related to sulfasalazine. All AEs that occur after consenting to the trial must be logged in detail in the CRF or recorded in an AE log. In the case of an AE which may be related to the administration of the IMP, a medically-qualified Investigator will initiate any appropriate treatment(s), according to their clinical judgement.

The Site Leaders are responsible for the detection, documentation and follow-up of events experienced by volunteers (at their respective hospitals) during their participation in the study that meet the criteria and definitions as detailed below.

10.1 DEFINITIONS

This trial is being undertaken with the approval of the Mercy Health HREC and the TGA's CTN scheme. Adverse events will be managed according to the following definitions, recommendations and requirements, guided by ICH-GCP principles, TGA guidelines (*19*), and the National Health and Medical Research Council (NHMRC)'s *National Statement (20)*.

An **Adverse Event** (AE) is any untoward medical occurrence in a study participant who has been administered an IMP. An AE <u>*does not necessarily have a causal relationship*</u> with the IMP in question.

An **Adverse Drug Reaction** (ADR) is any unintended, noxious response to an IMP at any dose, where a causal relationship between the IMP and the reaction *cannot be ruled out*.

A **Serious Adverse Event** (SAE) or **Serious Adverse Drug Reaction** (SADR) is defined as any AE or ADR that:

- Results in the death of a participant;
- Is life-threatening (puts the participant at risk of death of the time of the event – it does not refer to an event which could have hypothetically ended in death, had the event been more severe);
- Requires inpatient hospitalisation, or prolongation/escalation of existing hospitalisation;
- Results in a congenital abnormality or birth defect; or
- Results in significant or persistent incapacity/disability

An **Unexpected Adverse Drug Reaction** (UADR) is an ADR, the nature or severity of which is not consistent with the available manufacturer's Product Information (*Appendix 1).*

A **Suspected, Unexpected, Serious Adverse Reaction** (SUSAR) is any ADR that is:

- Classified as serious;
- Suspected to be caused by the IMP; and
- Not consistent with the information available in the manufacturer's Product Information

10.2 IDENTIFYING AEs AND SAEs

All AEs/SAEs will be recorded from the time a participant signs the consent form until their postnatal closing out visit or phone call has been completed. Worsening of preeclampsia disease indicators that follow the normal, expected clinical course of the disease will not be reported as an AE/SAE, but will be recorded in the CRF. Any AE must be recorded on the AE log page in the CRF. Antenatal admission to hospital will not be considered as an AE/SAE, as it follows the course of normal clinical management for this cohort. An escalation of hospitalisation (e.g. from general ward to intensive care) as a result of an ADR will be classified as a SAE/SADR.

Participants will be asked about the occurrence of AEs/SAEs at every research team contact episode during the study, using open-ended and non-leading questions (e.g. "Tell me about how you've been feeling"), as well as specific questions relating to any occurrence of common ADRs (e.g. gastrointestinal symptoms). Participants' medication charts will be reviewed to assess for study protocol compliance, as well as any major changes to concomitant medications. AEs/SAEs may also be identified via information from the medical history or other source documents (e.g. abnormal pathology test results not consistent with the woman's preeclampsia disease progression, such as aplastic anaemia). If there is any doubt whether or not an event or clinical observation constitutes an AE, the event will be recorded as an AE.

Known, common gastrointestinal side effects of sulfasalazine therapy (nausea, vomiting, diarrhoea) will be noted on the CRF but do not need to be recorded as AEs, unless meeting the criteria for a 'serious' or 'severe' event (see *Section 10.1*).

Some known, advanced side effects of sulfasalazine therapy (e.g. convulsions) may mimic signs and symptoms of worsening preeclampsia/eclampsia. It is not anticipated (based on the Manufacturer's Information – *Appendix 1*) that participants in this study will be receiving a high enough dose of sulfasalazine for long enough to develop these advanced side effects. Any occurrence of a convulsion in a study participant should be medically managed as a presumed eclamptic fit. However, because the relationship between any such event in a study participant taking sulfasalazine *cannot be absolutely excluded as a SADR/SUSAR*, it must still be recorded in the CRF, the AE log in the ISF and reported to the PIs, HREC and the TGA (as appropriate) as a SADR/SUSAR event (see *Sections 10.3-10.5*). The subsequent assessment of causality (see *Section 10.4.2*) may then deem the event to be an 'unlikely' SADR/SUSAR, and more probably the result of the progression of severe preeclampsia disease.

10.3 RECORDING AEs AND SAEs

Following notification of the occurrence of an AE or SAE, it is the responsibility of the relevant Site Leader to review all documentation and source data related to the incident, as well as recording and reporting the event to the relevant body/bodies (i.e. the PIs, HREC, and/or the TGA). The Site Leader must record all relevant information in the CRF and complete a SAE form, if the event meets the criteria of a 'serious' incident (as outlined in *Section 10.1*). Required information includes:

- Date of event onset
- Type of event
- Temporality
- Medication dosages
- The Site Leader's assessment of causality and severity
- Treatments/investigations required
- Time to resolution
- Incident outcome

These responsibilities may be delegated by a Site Leader to another suitably qualified medical doctor who is also a registered Investigator associated with the study (these responsibilities must be documented on the delegation log).

10.4 ASSESSMENT OF AEs AND SAEs

A medically-qualified Investigator must assess all AEs/ADRs as they occur for their seriousness, expectedness, causality and severity. As this is a single arm study, all AEs will be assessed knowing that the participant is taking the IMP. Any event that is classified as 'Serious' (SAE/SADR/SUSAR) must be notified to the PIs within 24 hours of a Co-Investigator's first awareness of the event.

The Site Leaders will review all AEs that are reported by the Co-Investigators as 'Serious' at their respective hospital sites. A Site Leader may not downgrade an event that has been assessed by another Investigator as 'serious' (SAE, SADR or SUSAR), but they may upgrade a reported AE/ADR to a 'serious event' as they deem appropriate. A PI, Site Leader, or the Trial Manager must also organise to inform the Mercy Health *Human Research Ethics Committee* (HREC) of any new information or research that comes to light during the trial regarding the safety of the IMP which could impact on the ethical acceptability of the study's continuation.

10.4.1 Assessment of seriousness

A medically-qualified Investigator who is named on the delegation log will make an assessment of any reported event's seriousness (as defined in *Section 10.1)*.

10.4.2 Assessment of causality

A medically-qualified Investigator will make an assessment of the likelihood of an AE/SAE being related to the IMP according to these definitions:

- 'Unrelated': Where an event is assessed as being not causally related to the administration of the IMP
- 'Possibly related': The AE is assessed as being possibly causally related to the IMP (based on the nature of the event, temporal relationship between AE and IMP dosing, concomitant medications, or the underlying medical condition). This assessment of causality will be referenced against the safety information detailed in the manufacturer's Product Information (*Appendix 1*). The Investigator may further sub-classify this assessment as 'unlikely, but possibly related', or 'likely to be related', according to their assessment of the event

Note: If an AE is considered to be attributed to the interaction between the IMP *and* another concomitant medication - or where an AE could be linked to *either* the IMP or a concomitant medication - the AE must be categorised as an ADR of sulfasalazine.

10.4.3 Assessment of expectedness

If an event is classified as an ADR, the assessing Investigator must use the information in the manufacturer's Product Information to assign a level of expectedness to the ADR, based on existing knowledge of the drug's potential to cause that particular event. The ADR must be termed as either:

- 'Expected': The ADR is consistent with the known toxicity profile of the IMP, as outlined in the manufacturer's Product Information (see *Appendix 1*); or
- 'Unexpected': The ADR is not consistent with the known toxicity profile of the IMP

10.4.4 Assessment of severity

The assessing Investigator will assign one of the three following categories of severity for each AE/SAE, and record their findings on the CRF/AE form:

- 'Mild': An AE that is easily tolerated by the participant, causing minimal discomfort and does not interfere with normal, daily activities
- 'Moderate': An AE that causes sufficient discomfort and interferes with the participant's normal, daily activities
- 'Severe': An AE that prevents the participants from being able to perform their normal daily activities

Note: 'Severity' and 'seriousness' should not be confused – a severity rating is used to describe the intensity of an event, while seriousness is a regulatory definition based on participant or event outcome (see *Section 10.1*). One AE might be serious but not severe (e.g. thrombocytopaenia), while another could be severe but not serious (e.g. nausea).

10.5 REPORTING OF SAEs/SADRs/SUSARs

Once an investigator becomes aware that a study participant has experienced a SAE/SADR/SUSAR, the information will be immediately reported to the CPI (no later than 24 hours after the assessing Investigator's first awareness of the event).

If an Investigator does not have detailed or complete information regarding the SAE/SADR/SUSAR, they should not delay informing the CPI about the event – once additional information is received, the SAE report form can be updated. The first submission of a SAE report form will outline an assessment of seriousness, causality, expectedness and severity (see *Sections 10.4.1- 10.4.4*) at the time of the initial report being made to the PIs.

SAE report forms can be submitted by hand to the co-ordinating trials office; transmitted by fax to **+61 (3) 8458 4380**; or sent in PDF format by email to amiddleton@mercy.com.au (Trial Manager); fiona.brownfoot@unimelb.edu.au (PI); **and** stong@unimelb.edu.au (PI). If a report is being submitted outside of business hours, phone contact must also be made with the on-call PI. Where missing information is not subsequently sent through to the Trial Manager/PIs following an initial report, the relevant investigator will be contacted to follow up and obtain additional information. All SAE reports and follow up information will be retained and compiled by the relevant Site Leader (or designee) in the *Investigator Site File* (ISF).

The PIs and Trial Manager will organise onward reporting to HREC and/or the TGA (as appropriate) following any SAE/SADR/SUSAR experienced by a study participant during this trial, as outlined in *Sections 10.6*, *10.7* and *Appendix 2*.

10.6 REGULATORY REPORTING REQUIREMENTS

The following table (TABLE 3) and *Appendix 2* from the TGA (*19*) summarises the reporting requirements for sponsors and clinical trial investigators relating to SAEs/SADRs/SUSARs in CTN/CTX scheme trials:

TABLE 3: TGA reporting requirements summary for serious events in clinical trials under CTN/CTX schemes:

Therapeutic Goods Administration

Reporter	→ Reports what?	→ To whom?	→In what format?	→ In what timeframe?
Sponsor of trial	Serious and unexpected adverse drug reactions	TGA*	ADRAC blue card$	For fatal or life-threatening ADRs, send initial report within 7 calendar days of first knowledge. Follow up with complete report within 8 additional calendar days.<br><br>For all other serious and unexpected ADRs, full report no later than 15 calendar days of first knowledge by the sponsor.
	Other reactions and adverse events	TGA	Tabulation	On request by TGA.
Clinical investigator(s)	Adverse reactions/events	HREC	As required by HREC	As required by HREC
		Sponsor of trial	As per study protocol	As per study protocol

* Report should be clearly marked 'Clinical trial ADR' and sent to:

The Medical Adviser
Experimental Drugs Section
Drug Safety and Evaluation Branch
Therapeutic Goods Administration
PO Box 100
WODEN ACT 2606

$ Or an appropriate format that contains the same information

As outlined in *Section 10.5*, Clinical Investigators must inform the PIs within 24 hours of their first knowledge of an AE that meet the criteria as being either 'serious' (as defined in *Sections 10.1* and *10.2*) and/or 'unexpected' (as outlined in *Sections 10.1* and *10.4.3*). The PIs and Trial Manager will initially inform HREC and the TGA of any SADRs/UADRs/SUSARs within 7 calendar days of their first knowledge of the event, with a full follow-up report due within 15 calendar days of their first knowledge of the event (as outlined in *TABLE 3*).

Other AEs/ADRs should be reported by the Co-Investigators in the CRF and forwarded to the PIs and Trial Manager in a timely manner. The PIs and Trial Manager will inform HREC of any events that meet the reporting requirements outlined by HREC in their conditional approval of the study.

10.7 FOLLOW UP PROCEDURES

After recording an AE on the CRF (or recording and reporting a SAE), the event will be followed by the research team until a final outcome can be recorded. This may include follow up visits, assessments or other contact with the affected participant that are supplementary to the course of normal clinical care.

If a resolution to an event cannot be established by the Investigators, an explanation of the efforts made to do so will be recorded in the CRF or AE log; or as an addition to the SAE reporting form submitted to HREC and the PIs.

Follow-up information from any SAEs will be reported as updates to the initial report form and submitted by the PIs within regulatory timeframes to the TGA and/or the local HREC (as appropriate).

11 PREGNANCY

There are no known contraindication(s) of sulfasalazine therapy to the current or subsequent pregnancies.

12 TRIAL MANAGEMENT AND OVERSIGHT ARRANGEMENTS

12.1 TRIAL STEERING COMMITTEE

The Trial Steering Committee (TSC) is made up of the study's Principal Investigators:

- Stephen Tong - Head of the University of Melbourne Translational Obstetrics Group/Co-director of Mercy Perinatal/Consultant Obstetrician
- Fiona Brownfoot - Co-ordinating Principal Investigator/Mercy Hospital for Women Site Leader/Consultant Obstetrician/MFM Fellow

- Joanne Said – Head of Maternal Fetal Medicine/Sunshine Hospital Site Leader/Consultant Obstetrician/CMFM

Along with Associate Researchers/Co-Investigators:

- Anna Middleton – Trial Manager/Mercy Perinatal Research Midwife
- Susan Walker - University of Melbourne Head of Department of Obstetrics and Gynaecology/Consultant Obstetrician/CMFM
- Tu'uhevaha Kaitu'u-Lino – University of Melbourne Translational Obstetrics Group Senior Scientist

The TSC is responsible for the protocol creation; the conduct and direction of the study in accordance with ICH-GCP principles and the approved study protocol; analysis of study data; oversight of the recruitment and treatment of trial participants; supervision of and delegation of roles to Co-Investigators; and communicating with the local HRECs, the TGA and the Data and Safety Monitoring Committee. The TSC will meet before the commencement of recruitment and thereafter on a 6-monthly basis throughout the conduct of the trial (or more frequently, as required).

12.2 DATA AND SAFETY MONITORING COMMITTEE

The Data and Safety Monitoring Committee (DSMC) will provide independent supervision for the trial. The DSMC will provide advice to the Sponsor, Investigational Sites, TSC and other Associate Researchers on all aspects of the trial, as well as providing protection for the participants by ensuring that the study is being run in accordance with ICH-GCP guidelines. The DSMC will specifically monitor for ongoing safety of trial participants and the scientific validity and merit of the trial. The DSMC will be made up of University of Melbourne-associated academic clinicians - a specialist neonatologist (Jim Holberton); an obstetric clinician-scientist (Stefan Kane); and a Maternal Fetal Medicine specialist (Elizabeth McCarthy). The DSMC will meet on a 6-monthly basis with representative(s) of the TSC throughout the conduct of the trial (or more frequently, as required).

12.3 INSPECTION OF RECORDS

All study Investigators (and other relevant employees) associated with the University of Melbourne, Mercy Hospital for Women and/or Sunshine Hospital will permit trial-related monitoring and audits to be undertaken by the DSMC; the TSC; local HRECs; and any regulatory authority inspections. In the case of an audit, trial monitoring activity, or regulatory inspection, all Investigators involved with the study agree to allow the inspectors and representatives of the Sponsor/HREC direct access to all study records and source data. This may include facilitating appropriate access to medical histories through the investigational sites' Health Information Services departments.

12.4 RISK ASSESSMENT

Specific interim risk assessments (encompassing clinical outcomes for mothers and babies enrolled in the study; gestation at delivery; and AE examinations) will be performed by the Trial Steering Committee and Data and Safety Monitoring Committee at meetings held every 6 months throughout the trial. These risk assessments will be performed to establish the need of any mid-trial study design adaptations and to provide information regarding the ongoing safety and ethical acceptability of the trial.

A summative risk assessment at the completion of the trial will help to inform one of the primary outcomes of the study – the safety profile of sulfasalazine in a preeclamptic cohort (see *Section 2.1.1*). The outcomes of each risk assessment will form the basis of subsequent trial monitoring and audit plans. The assessments will also provide information to researchers about specific features to incorporate into future study designs to mitigate risks to participants. HREC will be informed of the outcome of each risk assessment (with the next scheduled HREC update, if no major findings are made).

12.5 STUDY MONITORING AND AUDIT

The PIs, Trial Manager, or other designated representatives of the TSC will perform study monitoring activities in accordance with the trial monitoring plan (see *Section 12.6*). This will involve investigational site visits and central monitoring activities. Study audits will be performed by members of the TSC in accordance with the study audit plan, including investigator site, study documentation, study management and facility audits as necessary. The DSMC, HREC and/or TGA may also elect to undertake quality management or quality assurance activities to ensure the safe and ethical conduct of the trial and adherence to the study protocol.

12.6 TRIAL MONITORING PLAN

Study monitoring will be conducted at each Investigational Site during the trial for quality monitoring and assurance purposes. Member(s) or representative(s) of the TSC will perform audit activities at each site within 3 months of the commencement of recruitment, and every 6 months thereafter, for the duration of the data collection component of the study. Audit activities may include (but are not limited to) the inspection of consent forms to ensure appropriate completion; comparison of CRFs to source documents to ensure accuracy of transposed data; and discussions with Investigational Site staff regarding the conduct of the trial. Audit and monitoring results will be reported to HREC immediately if major breaches or issues are detected; otherwise with routine HREC updates that are scheduled to occur annually.

13 GOOD CLINICAL PRACTICE

13.1 ETHICAL CONDUCT

This study has been planned and will be implemented and conducted in accordance with ICH-GCP, TGA and NHMRC guidelines. The Researchers involved with this trial all hold current GCP certification; are currently undergoing GCP training; or work under the guidance of a GCP-certified fellow Investigator. The study has been granted conditional approval by Mercy Health HREC and RWH HREC to be conducted at Mercy Hospital for Women and Sunshine Hospital respectively.

13.2 REQUIRED APPROVALS AND CONDITIONS OF APPROVAL

Prior to study commencement, approval has been obtained for conducting the trial from Mercy Health HREC; ANZCTR; and the TGA (via the CTN scheme). Approval in kind has been granted for the conduct of the study at Sunshine Hospital by RWH HREC, who have deferred to Mercy Health's HREC approval and oversight of the study. Any conditions of approval have been met prior to the commencement of the study.

13.3 REGULATORY COMPLIANCE

Regulatory approval for conducting this trial has been gained from the TGA under the CTN scheme prior to the commencement of any study-specific procedures. The protocol and study conduct will comply with the NHMRC's *National Statement* (*20*) and the TGA's *Access to unapproved therapeutic goods in Australia (19)*.

13.4 INVESTIGATOR RESPONSIBILITIES

The PIs and Site Leaders retain overall responsibility of the conduct of the trial at their respective study sites; compliance with the study protocol; supervision of other researchers; and any protocol amendments. In accordance with ICH-GCP principles, the PIs are also responsible for the study components as described in *Sections 13.4.1-13.4.8*; however these responsibilities may be delegated to other appropriately-qualified researchers. Dissemination of these responsibilities will be appropriately documented in the delegation log for each site.

13.4.1 Informed consent

A registered Investigator associated with the SIP trial will approach women who are identified as eligible, potential participants to have an initial discussion about the trial, and to provide appropriate, HREC-approved PICFs. The (potential) participants must be made aware that their decision to take part in clinical research is completely voluntary in nature, and should be based on a clear and accurate understanding of what is involved, the potential risks and possible benefits of participating in the study. Any patients who ultimately consent to the trial will retain their copy of the PICF for their personal records.

Participants must receive adequate oral and written information about the study before being asked to provide their written, informed consent. The oral explanation must address all of the key elements outlined in the PICF. Given that this trial involves the use of an investigational medication, a PI or Co-Investigator, who is also a qualified obstetrician/obstetric trainee, is ultimately responsible for obtaining written, informed consent from each participant prior to the commencement of any protocol-specific procedures or treatments. When a Co-Investigator who is not a qualified obstetrician/obstetric trainee has contributed to the consenting process, their involvement will be appropriately documented on the CRF and the consent form.

Every opportunity must be given to the participants to ask questions; clarify any information that they do not understand; consult with any chosen family members/friends/treating clinicians; and seek any additional answers that they may need. The participant must be given sufficient time to consider and reflect upon the information provided. It should be emphasised that the participant may withdraw their consent at any point in time without compromising their access to clinical care, their relationship with the clinical care team, or their treating hospital.

As a part of the consenting process, participants must be informed and agree to their medical records being reviewed by relevant authorities or Sponsor personnel for regulatory inspection, trial monitoring and trial audit purposes. It should be made clear to the participant that their name and other personal details will not be disclosed outside of their treating hospital.

The Investigator(s) involved in the consenting process and the participant must each personally sign and date the informed consent form to confirm that participant's consent has been appropriately obtained. The signing of the consent form is a formal declaration by each party of their belief that the participant has a clear and accurate understanding of the trial; its purpose; what is involved; the potential risks and possible benefits; and with this knowledge, is readily volunteering for the study. The participant will be given a copy of their signed consent form for their personal records. Another copy will be filed in the participant's medical history, and the original copy will be stored in the ISF.

13.4.2 Study site staff

All of the study Investigators must be familiar with the IMP (sulfasalazine), study protocol, inclusion/exclusion criteria and the study requirements. It is the PIs' and Site Leaders' responsibility to ensure that all staff involved with the study at their respective hospitals have a clear understanding of the study protocol, their trial related duties, and the IMP.

13.4.3 Data recording

The PIs and Site Leaders are responsible for the quality of the data recorded in the CRF at their respective investigational sites. All relevant data captured by the CRFs will be transferred into a username and password-protected REDCap database by delegated Investigators, which will be created by the Trial Manager and CPI at Mercy Hospital for Women.

13.4.4 Investigator documentation

Before commencing work on the study, each member of the research team will be required to provide particular essential documentation to the Trial Manager and Mercy Health HREC to establish their credentials and suitability for the role. This documentation may include but is not limited to:

- A summarised Curriculum Vitae
- A signed 'Investigator's Declaration' form
- Evidence of ICH-GCP training/certification (if applicable)

The Trial Manager will ensure that all Investigator documents required by ICH-GCP guidelines are retained in a Trial Master File (TMF), with copies made available in the ISF at each participating hospital. The Trial Manager may delegate responsibility for the TMF to another appropriate researcher – this must be documented in the trial delegation log.

13.4.5 GCP training

All staff involved with the study must have undergone recent ICH-GCP training and hold current ICH-GCP certification of a level appropriate to their role; or be working under the close supervision of an Investigator who has.

13.4.6 Confidentiality

All staff involved with the study must comply with their hospital's local SOPs and protocols pertaining to confidentiality when undertaking work relating to this study. Additionally, to protect the confidentiality of trial participants and research staff members, the following steps will be taken:

- All participants will be allocated a unique study code, once informed consent has been obtained
- All source data transcriptions or copies, laboratory specimens, CRF entries, evaluation forms, reports and other records will be de-identified of participants' personal information and marked with their unique study code
- All participants' study records and staff information (e.g. CVs) will be kept in secure computer, work or storage areas with limited access
- Clinical information will not be shared with anyone outside of the research team or the participant's direct clinical carers, without the written permission of the participant (with the exception of any regulatory audit or quality management requirements)

13.4.7 Intellectual Property

Members of the research team will have a level of access to raw data, analysed data, study results, research records, and other unpublished and confidential information relating to this study through the course of their work. Without the express agreement of the PIs, no unpublished or confidential data obtained for this study may be:

- Used in any other capacity
- Used for any other research projects
- Disclosed to any third parties (with the exception of regulatory compliance activities)
- Published or presented in any capacity

13.4.8 Data protection

All members of the research team, investigational site staff and organisations involved in this study must comply with the requirements for the handling of participants' personal information set out in their local hospital's SOP and the Federal Australian *Privacy Act 1988* , with specific regard to the rules governing the collection, storage, disclosure and processing of health information for medical research.

Access to collated participant data will be restricted to relevant members of the research team; those clinicians involved in the direct clinical care of the participants in question; authorised representatives of the Sponsor; and any relevant regulatory authority personnel.

Computers and computer programs used to collect, collate and analyse data will have limited access measures (username and password protection). Any paper copies of data will be kept in secure, lockable filing cabinets in the trial site offices. Following the study, all data will be sent to secure, long term storage (as outlined in *Section 14.4*).

Published results of this study will be in statistical or tabulated form and will not contain any personal information that could lead to the identification of individual participants.

14 STUDY CONDUCT RESPONSIBILITIES

14.1 PROTOCOL AMENDMENTS

Any proposed changes to research activity or study protocol amendments relating to this trial must be reviewed and approved by the TSC and HREC, with the exception of those changes which are deemed necessary as an urgent safety measure or to remove an apparent threat to the immediate health or safety of a participant.

Proposed amendments to the study protocol must be submitted in writing for approval to Mercy Health HREC prior to any participants being enrolled under the amended protocol.

14.2 PROTOCOL VIOLATIONS AND DEVIATIONS

Protocol waivers, or prospective protocol deviations, will not be approved for this study (except where it is necessary to eliminate an immediate hazard to study participants). If changes to the study protocol are deemed necessary, they must be proposed as protocol amendments and submitted to HREC (as outlined in *Section 14.1*) for approval.

Any protocol deviations or violations that do occur will be recorded in a protocol deviation log at each investigational site. Each deviation must be reported to the PIs and Trial Manager within 24 hours of a researcher becoming aware that the deviation has taken place. The PIs and Trial Manager must compile the deviation logs and submit them for review to HREC.

14.3 SERIOUS BREACH REQUIREMENTS

A 'serious breach' is a protocol violation that is likely to cause a significant threat to either:

- The physical or mental health, safety or wellbeing of one or more trial participant(s); or
- The scientific merit, validity, conduct, or ethical acceptability of the trial

If any member of the research team becomes aware of a potential or suspected serious breach occurring, they must notify the on-call PI <u>*within 24 hours of their discovery of the information*</u>. It is the responsibility of the PIs, in consultation with the Trial Manager; other members of the Trial Steering Committee; and/or Data and Safety Monitoring Committee (as appropriate) to determine whether or not the event constitutes a 'serious breach'; to assess the potential or real impact of the breach on the validity of the trial or safety of participants; and to decide whether immediate reporting of the incident to HREC and/or any regulatory authorities may be warranted.

14.4 STUDY RECORD RETENTION

All documentation involved with this trial will be kept for a minimum of 25 years from the end of study date (as defined in *Section 14.5*), in accordance with TGA guidelines (*21*) and as required by Victorian law. The TSC or Sponsor may elect to outsource the safekeeping of records to a secure, off-site storage facility. Once the minimum retention period has lapsed, permission will be sought from senior representatives of the Sponsor before any records are permitted to be shredded (hard copies) or deleted (electronic copies).

14.5 END OF STUDY

The 'end of study' for this trial is defined as the last point of direct patient contact between the research team and the participants for data collection purposes. This would likely be the date of the postnatal closing out visit or phone call made to the final remaining participant in the study.

However, the TSC, DSMC and/or the Sponsor have the right to terminate the trial at any time prior to this point for clinical safety or administrative reasons. The date of trial termination would then constitute the 'end of study' date.

The end of study date will be reported to HREC within 90 days of completion; or within 15 days if the trial is terminated prematurely. The Site Leaders will inform participants in the instance of a premature study closure and ensure that any appropriate follow up is arranged for all participants involved.

A summary report of the study will be disseminated within 1 year of the completion of the trial, as described in *Section 15.2*.

14.6 CONTINUATION OF DRUG FOLLOWING THE END OF THE STUDY

Not applicable – IMP administration will be ceased with the delivery of a participant's pregnancy or upon their withdrawal from the study.

14.7 INSURANCE AND INDEMNITY

The Sponsor (the University of Melbourne Department of Obstetrics and Gynaecology) and Investigational Sites (Mercy Hospital for Women and Sunshine Hospital) are responsible for ensuring that proper provisions have been made for insurance and indemnity to cover their own liability, as well as that of the Principal Investigators, Associate Investigators and the other members of the research team relating to their work on this study.

The separate investigational sites involved with the study (Mercy Hospital for Women and Sunshine Hospital) will each retain their normal duty of care responsibilities towards participants of the study as patients receiving treatment at their respective health services. The investigational sites will be liable for any clinical negligence or other forms of negligent harm inflicted upon participants through their health service. The Sponsor of this study requires the individual investigation sites to have their own insurance arrangements in place relating to these responsibilities.

The following arrangements are in place to fulfil the Sponsor's obligations for the provision of indemnity cover relating to the conduct of this trial:

- This study is being conducted at the Mercy Hospital for Women in accordance with the conditional approval of the NHMRC-registered Mercy Health HREC (NHMRC code: EC00230).

- Approval in kind has been granted by the Royal Women's Hospital (RWH) HREC (EC00259) for the conduct of the study at Sunshine Hospital (deferred to by Melbourne Health HREC (EC00243), given the specific expertise of the RWH HREC in overseeing the conduct of clinical trials involving pregnant women).

- The conduct of this study has additionally been given regulatory approval by the ANZCTR (approval number ACTRN-1261-7000-2263-03) and by the TGA under the CTN scheme (submission number CT-2017-CTN-01723-1).

- The study protocol has been designed by Principal Investigator (Fiona Brownfoot) and Trial Manager (Anna Middleton) with the approval of Principal Investigator (Stephen Tong) and the University of Melbourne Head of Department of Obstetrics and Gynaecology (Susan Walker). The study protocol will be approved by the Trial Steering Committee, the Data and Safety Monitoring Committee and Mercy Health HREC prior to the commencement of the trial.

- A *Medicines Australia – Form of Indemnity for Clinical Trials (Standard)* will be co-signed by the Chief Executive of Health Services at MHVL (or their delegated representative); and the Head of Department of Obstetrics and Gynaecology at the University of Melbourne (Susan Walker) prior to the commencement of the trial. This form outlines the respective responsibilities for the provision of indemnity cover for researchers undertaking work on this study that is in accordance with the Mercy Health HREC-approved study protocol.

- A *Clinical Trial Research Agreement – Collaborative or Co-operative Research Group Studies Standard Form (CTRA)* will be completed prior to the commencement of recruitment to formalise the agreement for the conduct of the study between senior representatives of the Trial Steering Committee, MHVL and the University of Melbourne. This form will be co-signed by the study PI (Fiona Brownfoot); the Chief Executive of Health Services at MHVL (or their delegated representative); and the Head of Department of Obstetrics and Gynaecology at the University of Melbourne (Susan Walker).

- A CTRA will be completed to formalise the agreement for the conduct of the study between senior representatives of the Trial Steering Committee, Sunshine Hospital and the University of Melbourne. This form will be co-signed by PI (Joanne Said); Head of Department of Obstetrics and Gynaecology at the University of Melbourne (Susan Walker); and a senior Executive of Sunshine Hospital (or their delegated representative) prior to the commencement of recruitment at that site.

15 REPORTING, PUBLICATIONS AND NOTIFICATION OF RESULTS

15.1 AUTHORSHIP POLICY

Ownership of all data arising from this study remains with the members of the study team. Upon completion of the data collection component of the study, the data will be analysed (as outlined in *Section 9*), with an ICH-GCP-compliant clinical study report prepared.

15.2 PUBLICATION

The clinical study report will be submitted to the local HRECs, funding bodies (NHMRC, Norman Beischer Foundation and SOMANZ) and relevant regulatory authorities within one year of the study's completion. A copy will also be forwarded to senior representatives of the Sponsor and the Investigational Sites.

The study report will be used by approved members of the research team to present the results of the trial at scientific meetings. The PIs will have the right to submit/grant permission for the submission of the results of the study for written and/or oral publication.

A summary of the study results will be made available to the other members of the research team, who may, at their discretion, disseminate the findings within their clinics. A lay summary of the results will be made public on the Sponsor's and Investigational Sites' websites, in accordance with ICH-GCP recommendations. A lay summary may also be sent directly to the individual participants of the study, at the discretion of their respective hospital's Site Leader.

15.3 PEER REVIEW

This trial, its ethical acceptability and scientific merit was extensively reviewed by the members of Mercy Health HREC prior to the authorship of the study protocol or the commencement of study recruitment. The HREC panel includes academic, medical and midwifery specialists; ethicists; and lay-people.

REFERENCES

1. A. L. Tranquilli *et al.*, The classification, diagnosis and management of the hypertensive disorders of pregnancy: A revised statement from the ISSHP. *Pregnancy Hypertens* **4**, 97-104 (2014).
2. C. W. Redman, I. L. Sargent, Latest advances in understanding preeclampsia. *Science* **308**, 1592-1594 (2005).
3. B. Sibai, G. Dekker, M. Kupferminc, Pre-eclampsia. *Lancet* **365**, 785-799 (2005).
4. C. E. Powe, R. J. Levine, S. A. Karumanchi, Preeclampsia, a disease of the maternal endothelium: the role of antiangiogenic factors and implications for later cardiovascular disease. *Circulation* **123**, 2856-2869 (2011).
5. B. C. Young, R. J. Levine, S. A. Karumanchi, Pathogenesis of preeclampsia. *Annu Rev Pathol* **5**, 173-192 (2010).
6. T. Chaiworapongsa, P. Chaemsaithong, L. Yeo, R. Romero, Pre-eclampsia part 1: current understanding of its pathophysiology. *Nature reviews. Nephrology* **10**, 466-480 (2014).
7. ACOG, Report of the american college of obstetricians and gynecologists' task force on hypertension in pregnancy. *Obstet and gynecol.* **122**, 1122-1131 (2013).
8. J. A. Hutcheon, S. Lisonkova, K. S. Joseph, Epidemiology of pre-eclampsia and the other hypertensive disorders of pregnancy. *Best Pract Res Clin Obstet Gynaecol* **25**, 391-403 (2011).
9. S. Maynard, Min, JY, Merchan, J, Lim, KH, Li, J, Mondal, S, Libermann, TA, Morgan, JP, Sellke, FW, Stillman, IE, Epstein, FH, Sukhatme, VP, Ananth Karumanchi, S., Excess placental soluble fms-like tyrosine kinase 1 (sFlt-1) may contribute to endothelial dysfunction, hypertension, and proteinuria in pre-eclampsia. *The Journal of Clinical Investigation* **111**, 649-658 (2003).
10. S. E. Maynard, S. A. Karumanchi, Angiogenic factors and preeclampsia. *Semin Nephrol* **31**, 33-46 (2011).
11. T. Nagamatsu *et al.*, Cytotrophoblasts up-regulate soluble fms-like tyrosine kinase-1 expression under reduced oxygen: an implication for the placental vascular development and the pathophysiology of preeclampsia. *Endocrinology* **145**, 4838-4845 (2004).
12. S. Venkatesha *et al.*, Soluble endoglin contributes to the pathogenesis of preeclampsia. *Nat Med* **12**, 642-649 (2006).
13. T. G. Administration. (Canberra: Department of Health – Therapeutic Goods Administration, 2016).
14. S. Hahn, Preeclampsia - will orphan drug status facilitate innovative biological therapies? *Front Surg* **2**, 7 (2015).
15. B. W. Mol *et al.*, Pre-eclampsia. *Lancet* **387**, 999-1011 (2016).
16. W. H. Organization. (2016).
17. W. H. Organization, World Health Organisation recommendations for prevention and treatment of pre-eclampsia and eclampsia. (2011).
18. M. M. Costantine *et al.*, Safety and pharmacokinetics of pravastatin used for the prevention of preeclampsia in high-risk pregnant women: a pilot randomized controlled trial. *Am J Obstet Gynecol* **214**, 720 e721-720 e717 (2016).
19. TGA, *Access to unapproved therapeutic goods: Clinical trials in Australia. Canberra: Commonwealth of Australia: Therapeutic Goods Administration*, (2004).
20. NHMRC, National statement on ethical conduct in Human Research 2007 [Updated May, 2015]. *Canberra: Commonwealth of Australia – the National Health and Medical Research Council; the Australian Research Council; and the Australian Vice-Chancellors' Committee.* .
21. TGA, Note for guidance on good clinical practice (CPMP/ICH/135/95) - Annotated with TGA comments. Canberra: Therapeutic Goods Administration., (2000).

APPENDIX 1: Manufacturer's (Pfizer Australia) Product Information – Salazopyrin (sulfasalazine)
Retrieved from: http://secure.healthlinks.net.au/content/pf/retriever.cfm?product=pfpsalaa10913

PRODUCT INFORMATION

SALAZOPYRIN® and SALAZOPYRIN® EN-TABS (sulfasalazine)

NAME OF THE MEDICINE

SALAZOPYRIN (sulfasalazine) 500 mg tablets.

SALAZOPYRIN EN-TABS (sulfasalazine) 500mg enteric coated tablets.

The structural formula of sulfasalazine is shown below:

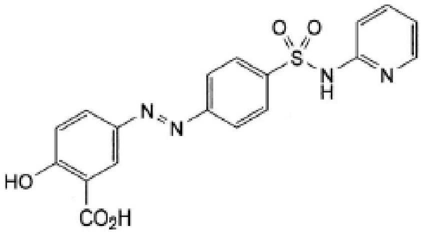

Chemical Name:	2-Hydroxy-5-[2-[4-(pyridin-2-ylsulphamoyl)phenyl]diazenyl]benzoic acid
Molecular Formula:	$C_{18}H_{14}N_4O_5S$
Molecular Weight:	398.4
CAS Registry Number:	599-79-1.

DESCRIPTION

Sulfasalazine is a bright yellow or brownish-yellow, fine powder. It is practically insoluble in water, very slightly soluble in ethanol (96 percent), practically insoluble in methylene chloride. It dissolves in dilute solutions of alkali hydroxides.

SALAZOPYRIN tablets contain 500 mg of sulfasalazine and the following inactive ingredients: starch-maize, silica-colloidal anhydrous, magnesium stearate, and povidone.

SALAZOPYRIN EN-TABS enteric coated tablets contain 500 mg of sulfasalazine and the following inactive ingredients: starch-maize, silica-colloidal anhydrous, magnesium stearate, cellacephate, propylene glycol, beeswax-white, carnauba wax, macrogol 20000, glyceryl monostearate, and talc-purified.

PHARMACOLOGY

Pharmacodynamics

Sulfasalazine has, among others, an immunosuppressive effect and has shown affinity to connective tissue. It has also been shown to have a wide range of effects in other biological systems. It is, however, difficult to judge the clinical relevance of its various pharmacological actions since the aetiology of rheumatoid arthritis is largely unknown. Moreover, the mode of action of sulfasalazine in the treatment of ulcerative colitis is also not known. A metabolite of the drug may have an inhibitory effect on an antigen-antibody process occurring in the intestinal wall and the salicylate component may act as an anti-inflammatory agent. The drug does not appear to have any long term antibacterial effect on the stool flora of patients with ulcerative colitis (see *"Antibacterial Effect"* below).

The following effects have been found *in vitro:* inhibition of bacterial growth; inhibition of prostaglandin synthesis; increased intestinal cytoprotection due to inhibition of prostaglandin degradation; reduction of leukotriene formation; modulation of polymorphonuclear leucocyte function; inhibition of proteolytic enzymes; inhibition of DNA synthesis; and impairment of folate absorption and metabolism.

Most of these effects have also been shown in experimental animal systems. This has led to the following alternative explanations of the clinical effects of sulfasalazine.

Antibacterial Effect

West *et al.*, (1974) found that the number of anaerobic bacteria in the colon lumen was markedly reduced by administration of sulfasalazine. Krook *et al.*, (1981) showed that 1 month of sulfasalazine treatment gave a drastic reduction in the anaerobic bacteria count. After four months of continuing treatment the counts returned to normal, although a decrease in certain bacterial strains was still evident.

Rheumatoid arthritis may be an enteropathogenic arthritis. One well known observation is that experimental arthritis in pigs may be caused by *Clostridium perfringens* in the large bowel. Neumann *et al.*, (1984) has recently examined the faecal flora in a normal control population and in rheumatoid arthritis patients during sulfasalazine treatment. During therapy there was a trend towards a decrease in the *Cl. perfringens* count in the sulfasalazine treated group which was not seen in the control group. Tremaine *et al.*, (1984) have recently shown that patients on sulfasalazine have a significantly lower incidence of *Cl. difficile* infection than patients with no treatment, and that a significantly higher incidence of infection is observed after antibiotic therapy.

Anti-inflammatory and Immunoregulatory Effect

In recent years a large number of papers, predominantly on *in vitro* studies, have reported effects of sulfasalazine on the arachidonic acid metabolites, prostaglandins and leukotrienes.

Any equivalence to these effects *in vivo*, seen as an anti-inflammatory effect, has yet only partly been documented. Thus, inhibition of carrageenan inflammation could be demonstrated in the rat paw and in the rat colon. In the adjuvant arthritis model in rats, Steinwall *et al.*, showed a significant inhibition at high doses of sulfasalazine. Indomethacin-induced ulceration could be reduced by sulfasalazine, and water transport was normalised by sulfasalazine both in human biopsy tissue and in the dinitrochlorobenzene (DNCB) colitis in a rat model.

With regard to possible immunoregulatory effects, Holm and Perimann (1968) published a study on the effect of sulfasalazine upon lymphocytic activity. Sulfasalazine was shown to have a specific toxicity for lymphocytes, higher than for other cells. Campbell (1973) studied the effect of sulfasalazine in immunological models, and a clear immunoregulatory effect was noted.

Laursen (1978) found that sulfasalazine in mice suppressed many immunological factors, such as serum levels of different immunoglobulins and the number of lymphocytes. Rubenstein *et al.*, (1978) and Ali *et al.*, (1982) have shown that sulfasalazine therapy depresses the activity of certain lymphocytes.

Pharmacokinetics

Absorption

After the administration of a single 2 g dose of SALAZOPYRIN (enteric or non enteric coated tablets), sulfasalazine can be detected in serum within 6 hours. Two days after administration the serum concentration is negligible. With repeated daily doses, the steady state serum level of sulfasalazine is achieved after 4 to 5 days.

Sulfasalazine is poorly absorbed from the small intestine (up to 30%), after which it is excreted into the bile. A few percent of the given dose is excreted unmetabolised into the urine. The unabsorbed drug is excreted in the faeces. This is also confirmed by the observation that around 70% of an ingested dose of sulfasalazine can be recovered in the effluent of ileostomised patients. Sulfasalazine rapidly enters the enterohepatic circulation and is returned to the gastrointestinal tract via the biliary route. Up to 80% of the absorbed intact sulfasalazine can be recovered in the bile effluent.

Metabolism

The 70 to 80% of the ingested dose of intact sulfasalazine which reaches the colon is subjected to azo-reductive cleavage by the colonic flora to yield the two major metabolites, 5-amino-salicylic acid (5-ASA) and sulfapyridine. Sulfapyridine is rapidly absorbed, partly metabolised in the liver, primarily by acetylation, and subsequently excreted in the urine. Non-acetylated sulfapyridine is partly protein bound. The role of the gut bacteria in splitting sulfasalazine has been elucidated in detail by Peppercorn and Goldman (1972). Thus, the delay in appearance of sulfapyridine and 5-ASA in the serum after oral administration of sulfasalazine is consistent with the time taken for the drug to reach the microbially rich colon. The two products of cleavage, 5-ASA and sulfapyridine are subsequently metabolised.

In humans only N-acetyl-5-aminosalicylic acid has been identified as a metabolite of 5-ASA. Sulfapyridine metabolites have been identified in humans as: N^4-acetylsulfapyridine; sulfapyridine-o-glucuronide; acetylsulfapyridine-o-glucuronide; 5-hydroxy-sulfapyridine-o-glucuronide; and N^4-acetyl-5-hydroxy-sulfapyridine-o-glucuronide.

The rate at which sulfapyridine is acetylated in the liver is genetically determined. Patients can be readily classified as slow or fast acetylator phenotypes on the basis of the ratio of sulfapyridine and acetylsulfapyridine concentrations in serum or urine. There is also a genetic basis for differentiation of patients according to the rate at which sulfapyridine is hydroxylated. Those subjects who show relative slowness in acetylation and hydroxylation rates may be expected to show higher than normal serum concentration of sulfapyridine after administration of sulfasalazine.

Distribution and Excretion

Intact sulfasalazine can be detected in serum within 6 hours after oral administration and between 2 and 10% of a single dose can be recovered as the unchanged molecule from the urine. No detectable sulfasalazine has been found in stools of normal subjects, although up to 7% of an orally administered sulfasalazine dose has been found in the faeces of patients having ulcerative colitis. Sulfasalazine and its metabolites excreted in the urine may impart an orange-yellow colour to alkaline urine.

Most of the 5-aminosalicylate moiety formed by bacterial action in the gut is excreted unchanged in the faeces. However, up to 33% of the salicylate absorbed can be recovered in the urine almost entirely as N-acetyl-5-aminosalicylic acid.

Both sulfasalazine and 5-ASA exhibit an affinity for collagen rich tissues. It has been shown that these two entities tend to concentrate primarily in the intestinal wall as well as in the peritoneal, pleural and synovial fluids. There is also evidence that blood borne sulfasalazine can enter the intestinal lumen directly from the serum, for it has been shown by Hannagren *et al*., (1973) that intravenously administered sulfasalazine concentrates intestinally in rats with ligated bile ducts.

The sulfapyridine formed by cleavage of sulfasalazine during azo-reduction by the intestinal flora is absorbed in the intestinal tract and appears to be evenly distributed in the various body tissues and fluids. About two thirds of the amount of sulfapyridine present in sulfasalazine is excreted in the urine, partially as acetylated or glucuronidated metabolites. The faeces contain sulfapyridine equivalent to approximately 7% of the ingested dose. There is no detectable sulfapyridine in serum 3 days after termination of treatment.

Protein Binding

Greater than 95% of absorbed sulfasalazine is bound to serum proteins.

INDICATIONS

Ulcerative Colitis and Crohn's Disease

Adjunct in the treatment of ulcerative colitis with the usual supportive and dietary measures. For the management of severe, acute attacks of ulcerative colitis, rectal and systemic corticosteroid therapy appears to be clinically superior to sulfasalazine, but sulfasalazine may be more effective than corticosteroids in reducing the number of relapses in patients on maintenance therapy.

In the treatment of active Crohn's disease, especially in patients with colonic involvement.

Rheumatoid Arthritis

SALAZOPYRIN EN-TABS are indicated for rheumatoid arthritis which has failed to respond to non-steroidal anti-inflammatory drugs (NSAIDs).

CONTRAINDICATIONS

Haematological, renal or hepatic dysfunction, allergic drug fever or skin eruptions due to sulphonamide derivatives including antibacterial sulphonamides, oral hypoglycaemics and thiazides.

Patients hypersensitive to sulfasalazine, its metabolites, or any other component of the product, sulfonamides, or salicylates.

Intestinal or urinary obstruction.

Patients with porphyria should not receive sulphonamides, as these drugs have been reported to precipitate an acute attack.

Children aged 2 years and younger.

PRECAUTIONS

Serious Infections[±]

Serious infections associated with myelosuppression, including sepsis and pneumonia, have been reported. Patients who develop a new infection while undergoing treatment with sulfasalazine should be monitored closely. Administration of sulfasalazine should be discontinued if a patient develops a serious infection. Caution should be exercised when considering the use of sulfasalazine in patients with a history of recurring or chronic infections or with underlying conditions which may predispose patients to infections.

Potential Toxicity

Deaths associated with the administration of sulfasalazine have been reported, resulting from hypersensitivity reactions, agranulocytosis, aplastic anaemia, renal and liver damage, irreversible neuromuscular and CNS changes, and fibrosing alveolitis. The presence of clinical signs such as sore throat, fever, pallor, purpura or jaundice may be indications of myelosuppression, hepatotoxicity, haemolysis or other serious blood disorders. The patient should be advised to report any untoward symptoms immediately. If serious toxic or hypersensitivity reactions occur, discontinue treatment with sulfasalazine immediately while awaiting the results of blood tests. Urticaria, other skin rashes and serum sickness-like reactions may be controlled with antihistamines and, if necessary, systemic corticosteroids.

Monitoring for Toxicity

Sulfasalazine should be administered under constant medical supervision.

Complete blood counts (including differential white cell count) and liver function tests should be performed before starting sulfasalazine tablets and every second week during the first three months of therapy. During the second three months, the same tests should be done once monthly and thereafter once every three months, and as clinically indicated. Assessment of renal function (including urinalysis) should be performed in all patients initially and at least monthly for the first three months of treatment. Thereafter, monitoring should be performed as clinically indicated.

Hepatic or Renal Impairment and Blood Dyscrasias

Sulfasalazine should not be given to patients with impaired hepatic or renal function or with blood dyscrasias.

Hypersensitivity

Sulfasalazine should be given with caution in patients with severe allergy or bronchial asthma.

Severe hypersensitivity reactions may include internal organ involvement, such as hepatitis, nephritis, myocarditis, mononucleosis-like syndrome (i.e. pseudomononucleosis), haematological abnormalities (including haematophagic histiocytosis), and/or pneumonitis including eosinophilic infiltration.±

Drug Rash with Eosinophilia and Systemic Symptoms (DRESS)±

Severe, life-threatening, systemic hypersensitivity reactions such as drug rash with eosinophilia and systemic symptoms (DRESS) have been reported in patients taking various drugs including sulfasalazine. It is important to note that early manifestations of hypersensitivity, such as fever or lymphadenopathy, may be present even though rash is not evident. If such signs or symptoms are present, the patient should be evaluated immediately. Sulfasalazine should be discontinued if an alternative etiology for the signs or symptoms cannot be established.

Serious Skin Reactions±

Serious skin reactions, some of them fatal, including exfoliative dermatitis, Stevens-Johnson syndrome, and toxic epidermal necrolysis, have been reported very rarely in association with the use of sulfasalazine. Patients appear to be at highest risk for these events early in the course of therapy, the onset of the event occurring in the majority of cases within the first month of treatment. Sulfasalazine should be discontinued at the first appearance of skin rash, mucosal lesions, or any other sign of hypersensitivity.

Effects on Folic Acid

Oral sulfasalazine inhibits the absorption and metabolism of folic acid and may cause folic acid deficiency, potentially resulting in serious blood disorders (e.g. macrocytosis and pancytopenia) and the possibility of harming the foetus during pregnancy (see Use in Pregnancy).

G-6-PD Deficiency

Patients with a deficiency of erythrocytic glucose-6-phosphate dehydrogenase (G-6-PD) have been noted to develop haemolytic anaemia during treatment with sulfasalazine (Cohen *et al.*, 1968; Gabor, 1973) and should be closely observed.

Fluid Intake

Adequate fluid intake must be maintained in order to reduce the risk of crystalluria and stone formation.

Patients with Atopic Disease

Sulfasalazine should be given with caution to patients with history of atopic disease in view of the increased likelihood of hypersensitivity reactions in atopic patients.

Interactions

Sulphonamides should be administered with caution to patients receiving other drug therapy (see INTERACTIONS WITH OTHER MEDICINES).

Reversible Male Infertility

Several recent reports have suggested that sulfasalazine may cause reversible infertility in males (Grieve, 1979; Levi *et al*., 1979; Toth, 1979; Traub *et al*., 1979; Toovey *et al*., 1981). Sulfasalazine therapy has been associated with a reduction in sperm counts, reduced sperm motility, morphologically abnormal sperm and an increased proportion of immature sperm. The mechanism by which sulfasalazine might affect sperm production is not understood. Until such time as this suggested association can be elucidated, a drug associated cause should be considered when investigating infertility in men taking sulfasalazine. Withdrawal of the drug usually reverses these effects within 2 to 3 months.

Use in Pregnancy

Pregnancy Category: A.

Oral sulfasalazine inhibits the absorption and metabolism of folic acid and may cause folic acid deficiency, potentially resulting in serious blood disorders (e.g. macrocytosis and pancytopenia) and the possibility of harming the foetus during pregnancy.

There have been reports of babies with neural tube defects born to mothers who were exposed to sulfasalazine during pregnancy, although the role of sulfasalazine in these defects has not been established. Because the possibility of harm cannot be completely ruled out, sulfasalazine should be used during pregnancy only if clearly needed.[±]

Use in Lactation

The amount of sulfasalazine that passes into the maternal milk is negligible, however, the concentration of sulfapyridine in milk is about 40% of that in serum. The risk of kernicterus in breast-fed infants has been assessed as low with therapeutic doses, since sulfapyridine has been shown to have a poor bilirubin displacing capacity.

As with all drugs, sulfasalazine should not be given to nursing mothers unless the expected benefits to the mother outweigh the potential risk to the infant. Caution should be used, particularly if breastfeeding premature infants or those deficient in G-6-PD. There have been reports of bloody stools or diarrhoea in infants of mothers on sulfasalazine who were breastfeeding.

Paediatric Use

Use in children with systemic onset juvenile rheumatoid arthritis may result in a serum sickness-like reaction; therefore, sulfasalazine is not recommended in these patients.

Effects on Laboratory Tests

Several reports of possible interference with measurements, by liquid chromatography, of urinary normetanephrine causing a false-positive test result have been observed in patients exposed to sulfasalazine or its metabolite, mesalazine.

INTERACTIONS WITH OTHER MEDICINES

Sulphonamides may potentiate oral anticoagulants, methotrexate, and oral hypoglycaemics of the sulphonylurea type by displacing these drugs from their binding sites.

An increased incidence of gastrointestinal adverse events, especially nausea, has been reported with co-administration of oral sulfasalazine and methotrexate to rheumatoid arthritis patients.

Increased sulphonamide blood levels may occur in patients who are receiving urinary acidifiers, oral anticoagulants, indomethacin or salicylates.

Antacids

Decreased absorption of sulphonamides from the gastrointestinal tract may occur if antacids are given concurrently.

Penicillins

It has been reported that sulphonamides interfere with oral absorption of oxacillin and may inhibit the serum protein binding of penicillins.

Local Anaesthetics Derived from Para-aminobenzoic Acid

Local anaesthetics which are derivatives of para-aminobenzoic acid may antagonise sulphonamide activity.

Digoxin

Reduced absorption of digoxin, resulting in non-therapeutic serum levels, has been reported when used concomitantly with sulfasalazine.

Folic Acid

Folate deficiency may occur as sulfasalazine inhibits the absorption of folate.

Ferrous Sulphate

Ferrous sulphate may impair the absorption of sulfasalazine however, the clinical significance of this interaction is doubtful.

Thiopurine 6-Mercaptopurine or Azathioprine

Due to inhibition of thiopurine methyltransferase (TPMT) by sulfasalazine, bone marrow suppression and leucopenia have been reported when thiopurine 6-mercaptopurine or its prodrug, azathioprine, and oral sulfasalazine were used concomitantly.

ADVERSE EFFECTS

Sulfasalazine shares the toxic potentialities of other sulphonamides, especially sulfapyridine, and the usual precautions of sulphonamide therapy should be observed. Moreover, it may be difficult to evaluate an adverse reaction in the individual case, since several of the untoward symptoms and signs encountered in conjunction with treatment with sulfasalazine may be part of the disease. In assessing liver and joint complications, it should also be borne in mind that such are often associated with ulcerative colitis.

The acetylation rate of sulfapyridine is determined by genetic factors. Slow acetylators can be expected to show higher serum levels of sulfapyridine, and thus may show an increased tendency towards adverse reactions.

Many side effects are dose dependent, and the symptoms can often be alleviated by reducing the dosage by greater subdivision of the dose.

The most common side effects are: nausea, vomiting and anorexia (which occur more frequently in patients receiving non-enteric coated sulfasalazine. To minimise the risk of gastrointestinal adverse reactions enteric coated tablets are used, which means that they do not disintegrate until they reach the small intestine); raised temperature; erythema and pruritus; headache; reversible oligospermia (oligospermia and infertility have been described in men treated with sulfasalazine. Withdrawal of the drug will reverse these effects).

The majority of the adverse reactions listed below have only seldom been reported, primarily in treatment of inflammatory bowel diseases and are typical of sulphonamides.

Infections and Infestations

Aseptic meningitis, pseudomembranous colitis.

Blood and Lymphatic System Disorders (see PRECAUTIONS)

Red cell abnormalities[#] (e.g. haemolytic anaemia, macrocytosis), aplastic anaemia, megaloblastic anaemia, pseudomononucleosis[*‡], hypoprothrombinaemia, methaemoglobinaemia, bone marrow depression with leucopenia (e.g. agranulocytosis, thrombocytopenia), pancytopenia.

Immune System Disorders

Serum sickness.

Hypersensitivity Reactions

Anaphylaxis*/anaphylactoid reactions, periorbital or facial oedema, conjunctival and scleral injection and nephrotic syndrome.

Metabolism and Nutrition Disorders

Anorexia, folate deficiency[*‡] (see PRECAUTIONS).

Psychiatric Disorders

Mental depression.

Nervous System Disorders

Dizziness#, smell and taste disorders, peripheral neuropathy, headache, peripheral neuritis, convulsions, hallucinations, vertigo, insomnia, transient lesions of posterior column and transverse myelitis, encephalopathy.

Ear and Labyrinth Disorders

Tinnitus#.

Cardiac Disorders

Myocarditis* (including allergic myocarditis) (see PRECAUTIONS)±, pericarditis, cyanosis#.

Vascular Disorders

Pallor* (see PRECAUTIONS)±.

Respiratory, Thoracic and Mediastinal Disorders (see PRECAUTIONS)

Lung complications (fibrosing alveolitis with e.g. dyspnoea, cough, eosinophilic infiltration), interstitial lung disease*, oropharyngeal pain*±.

Gastrointestinal Disorders

Gastric distress#, abdominal pain#, nausea, vomiting*, diarrhoea*, pancreatitis, stomatitis, impaired folic acid absorption, aggravation of ulcerative colitis*.

Hepatobiliary Disorders (see PRECAUTIONS)

Hepatic failure*, hepatitis fulminant*, hepatitis, jaundice*±, hepatitis cholestatic*±, cholestasis*±.

Skin and Subcutaneous Tissue Disorders (see PRECAUTIONS)

Toxic epidermal necrolysis (Lyell's syndrome), erythema multiforme, Stevens-Johnson syndrome, drug rash with eosinophilia and systemic symptoms (DRESS)*, toxic pustuloderma, alopecia, erythema, exanthema, exfoliative dermatitis, angioedema*, lichen planus, photosensitivity, purpura*, pruritus, urticaria, generalised skin eruptions.

Musculoskeletal and Connective Tissue Disorders

Systemic lupus erythematosus, Sjögren's syndrome, arthralgia.

Renal and Urinary Disorders

Proteinuria#, haematuria#, crystalluria# (see PRECAUTIONS), nephrotic syndrome, interstitial nephritis, nephrolithiasis*±.

Reproductive System

Reversible oligospermia (see PRECAUTIONS).

General Disorders

Fever (see PRECAUTIONS), yellow discolouration of skin and body fluids*, petechiae and drug fever, periarteritis nodosum and LE phenomenon have occurred.

Investigations

Induction of autoantibodies, elevation of liver enzymes.

# Adverse effects are possibly dose-related.

* Adverse effects identified post-marketing.

DOSAGE AND ADMINISTRATION

Inflammatory Bowel Disease

Oral: SALAZOPYRIN or SALAZOPYRIN EN-TABS (enteric coated) should be given preferably after meals in evenly divided doses over a 24 hour period with no more than 8 hours between overnight doses. The enteric coated tablets should not be crushed or broken.

Initial Dosage

Adults: 1 to 2 g four times daily.

Children: 40 to 60 mg/kg bodyweight daily in three to six divided doses.

Maintenance Dosage

Adults: 2 g daily in four divided doses.

Children: 40 mg/kg bodyweight daily in four divided doses. The daily maintenance dose should be continued unless contraindicated by side effects.

SALAZOPYRIN EN-TABS may be used to minimise gastrointestinal intolerance to the drug.

Rheumatoid Arthritis

Oral: Two SALAZOPYRIN EN-TABS, two or three times a day, i.e. 2 to 3 g daily. The enteric coated tablets should not be crushed or broken. For adults starting therapy, it is advisable to raise the daily dose according to the following schedule:

Adults

	1st WEEK	2nd WEEK	3rd WEEK	4th WEEK
Morning		1 tablet	1 tablet	2 tablets
Evening	1 tablet	1 tablet	2 tablets	2 tablets*
* etc. to 3 g/day maximum.				

Children: At present no dosage recommendation regarding treatment with SALAZOPYRIN EN-TABS in rheumatoid arthritis in children can be given.

OVERDOSAGE

The drug has low acute per oral toxicity in the absence of hypersensitivity. There is evidence that the incidence and severity of toxicity following overdosage are directly related to the total serum sulfapyridine concentration.

Signs and Symptoms

Similar to those of any sulphonamides. The most likely symptoms would be gastrointestinal disturbances (nausea, vomiting and abdominal pain), haematuria, crystalluria or anuria. In more advanced cases, central nervous system symptoms such as drowsiness, convulsions, etc., may be observed. Patients with impaired renal function are at increased risk of serious toxicity. There are no documented reports of deaths due to ingestion of large single doses of sulfasalazine.

Treatment of Overdosage

There is no specific antidote and treatment is symptomatic and supportive. Alkalinize urine (2.5 to 4.0 g of sodium bicarbonate every 4 hours). If kidney function is normal, force fluids. If anuria is present, restrict fluids and salt and treat for renal failure. Sulfasalazine can be removed by haemodialysis. Catheterisation of the ureters may be indicated for complete renal blockage by crystals. For agranulocytosis discontinue the drug immediately, hospitalise the patient and institute appropriate therapy. Patients should be observed for development of methaemoglobinaemia or sulfhaemoglobinaemia. If these occur, treat appropriately.

Contact the Poisons Information Centre on 13 11 26 for advice on the management of an overdose.

PRESENTATION AND STORAGE CONDITIONS

SALAZOPYRIN Tablets 500 mg

Yellow-orange, round, scored tablets; marked with `KPh' on the one side and `101' on the other side.

These tablets are supplied in bottles of 100 tablets.

SALAZOPYRIN EN-TABS 500 mg

Yellow-orange, elliptical convex, enteric coated tablets; marked with `KPh' on the one side and `102' on the other side.

These tablets are supplied in bottles of 100 tablets.

Storage

SALAZOPYRIN tablets and SALAZOPYRIN EN tablets should be stored below 25°C.

NAME AND ADDRESS OF THE SPONSOR

Pfizer Australia Pty Ltd
ABN 5000 8422 348
38-42 Wharf Road
WEST RYDE NSW 2114.

POISON SCHEDULE OF THE MEDICINE

Schedule 4 – Prescription Only Medicine.

DATE OF FIRST INCLUSION IN THE AUSTRALIAN REGISTER OF THERAPEUTIC GOODS

05 September 1991.

DATE OF MOST RECENT AMENDMENT

17 September 2013.

® Registered trademark.

= Please note change(s) to product information.

References

Ali, A.T.M.M. *et al.*, (1982): Lancet I: 506-7.

Campbell, D.E.S. (1973): Lakartidningen 70: 3068-3071.

Cohen, S.M. *et al.*, (1968): J. Amer. Med. Ass. 205: 528-530.

Gabor, E.P. (1973): N. Engl. J. Med. 289: 1372.

Grieve, J. (1979): Lancet I: 464.

Hannagren, A. (1973): Acta Med. Scand. 173: 61-72, 391-9.

Holm, G. and Perimann, P. (1968): In Advances in Transplantation (Ed. Dausset, J. *et al.*) Munksgaard, Copenhagen, pp 719-730.

Krook, A. *et al.*, (1981): Scand. J. Gastroenterol. 16: 183-192.

Laursen, M.L. (1978): Scand. J. Gastroenterol. 13: 991-7.

Levi, A.J. *et al.*, (1979): Lancet II: 276-278.

Neumann, V.C. *et al.*, (1984): J. Royal. Soc. Med. 77: 169-72.

Peppercorn, M.A. and Goldman, P. (1972): J. Pharmacol. Exp. Ther. 181: 555-562.

Rubenstein, A. *et al.*, (1978): Clin. Exp. Immol. 33: 217-224.

Toovey, S. *et al.*, (1981): Gut 22: 445-451.

Toth, A. (1979): Fertility & Sterility 31: 538-540.

Traub, A.I. *et al.*, (1979): Lancet II: 639-640.

Tremaine, W.J. *et al.*, (1983): Gastroenterology 84: 1337.

West, B. *et al.*, (1974): Gut 15: 960-5.

APPENDIX 2: Event reporting algorithm for sponsors
From TGA's *Access to unapproved therapeutic goods in Australia* (2004)

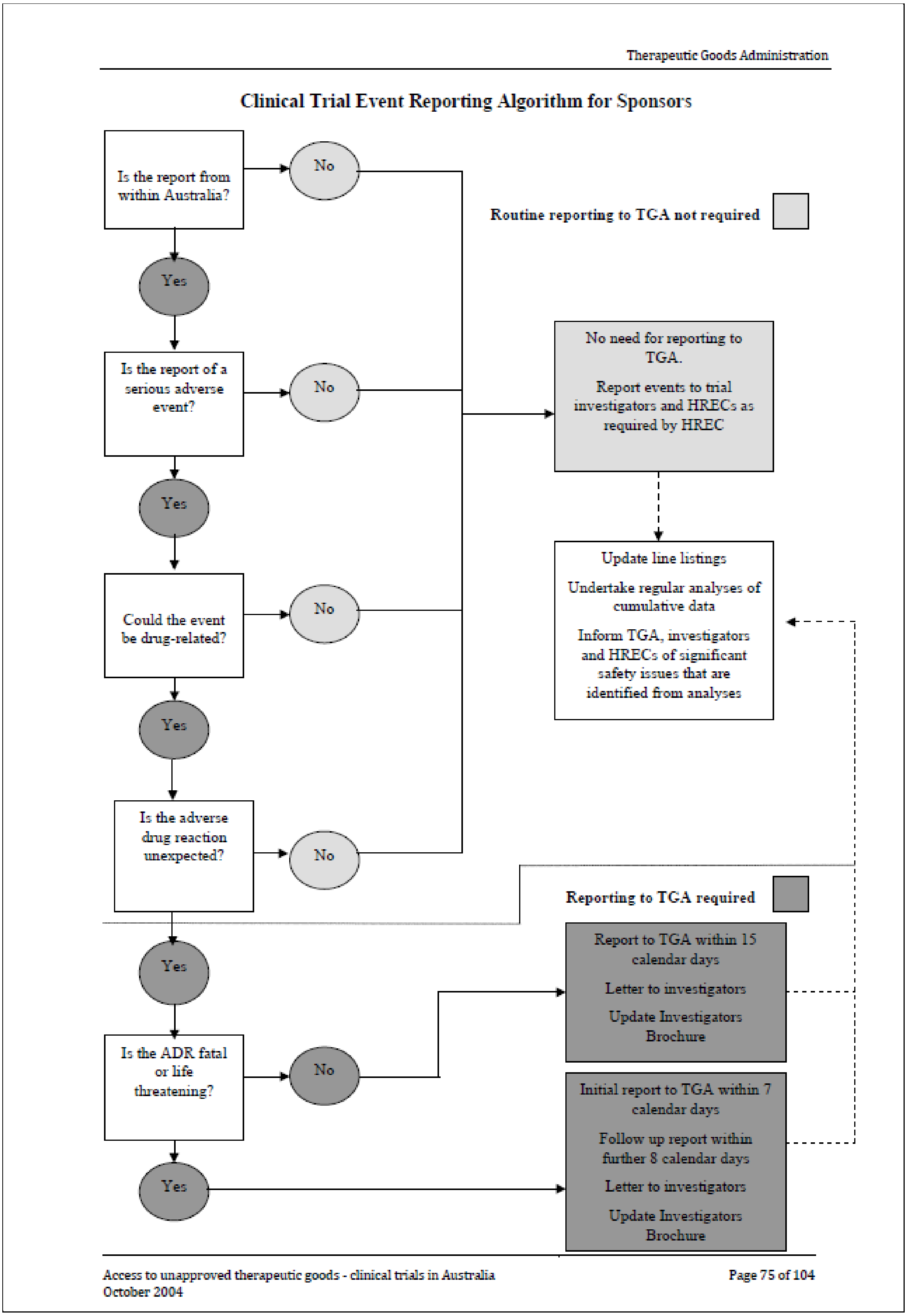

Supplement: SIP trial protocol [file mmc1.pdf]
